# Stimuli-responsive vesicles in water-resistant nanofibers for molecular random access in DNA data storage

**DOI:** 10.1039/d6na00595k

**Published:** 2026-09-21

**Authors:** Jokin Yeregui-Elosua, Diana Soukarie, Cecilia Wetzl, Alexander M. Bittner, Ibon Santiago

**Affiliations:** a CIC NanoGUNE BRTA Donostia-San Sebastián 20018 Spain i.santiago@nanogune.eu; b University of the Basque Country, EHU Donostia-San Sebastián 20018 Spain; c IKERBASQUE, Basque Foundation for Science 48009 Bilbao Spain

## Abstract

DNA is an attractive medium for data storage. Enabling efficient random access is essential for the practical use of DNA-based memory. We present a strategy for random access based on the selective release of target DNA from a nanofiber mesh matrix. Messages encoded in sets of DNA sequences are encapsulated inside stimuli-responsive small unilamellar vesicles (SUVs), embedded in an electrospun water-resistant nanofiber mesh. The target sequences are selectively released using orthogonal pH-based triggers. The orthogonality of these triggers enables the preservation of the SUVs containing the non-selected DNA, which remains in the fiber mesh for subsequent retrieval. This work combines sequence-independent chemical addressing with physical compartmentalization and double-layered protection. It represents initial steps towards a PCR-independent molecular selection system for random access in water-resistant fibers with potential applications in DNA-based memory architectures.

## Abstract 2

Sequential selective retrieval of data-encoding DNA *via* orthogonal pH-responsive lipid vesicles embedded in cross-linked PEO electrospun fibers, combining sequence-independent chemical addressing with physical separation and double-layer protection.

## Graphical abstract

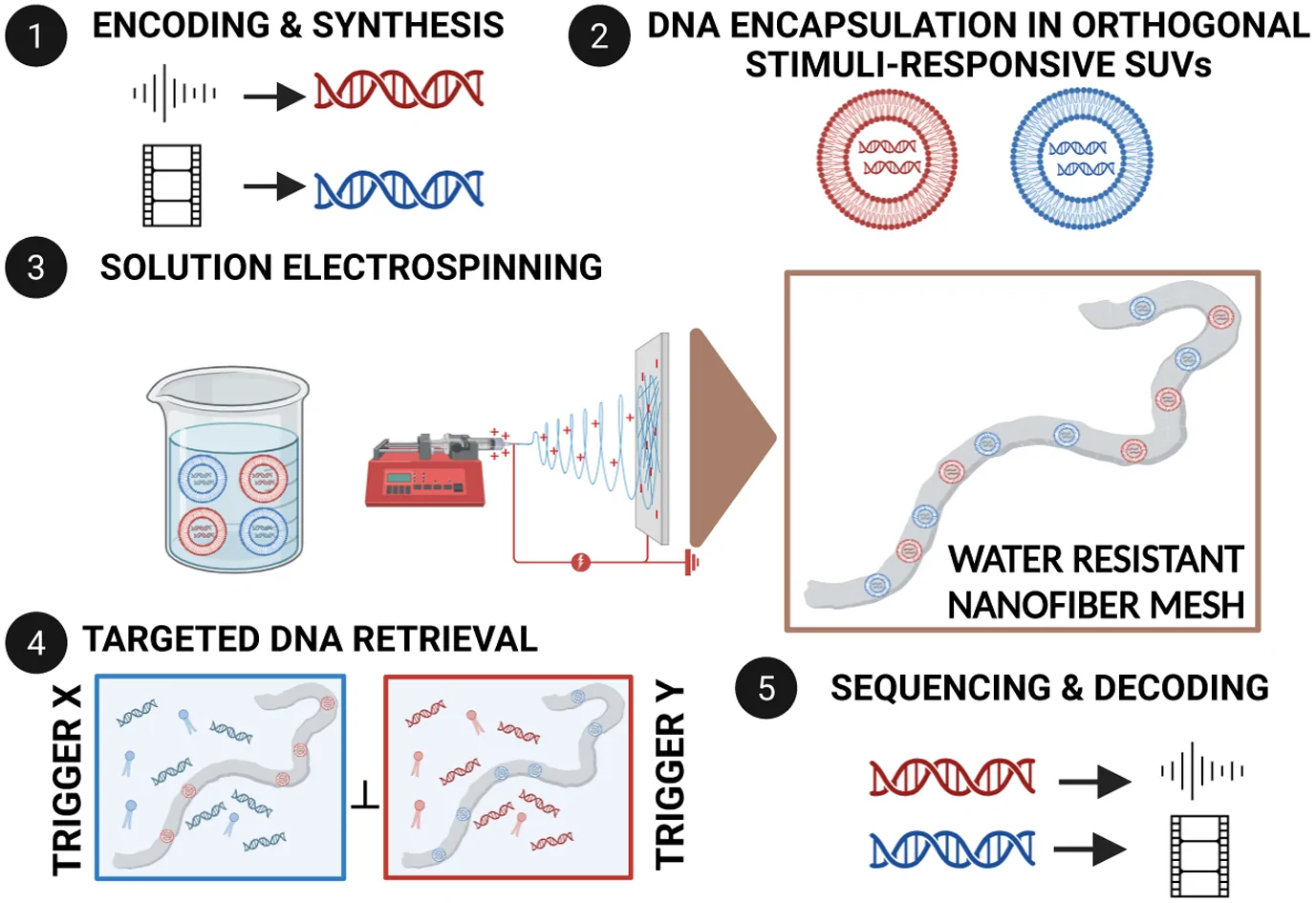

## Introduction

DNA has proven to be a promising platform for long-term digital data storage due to its high information density, stability, and durability.^1,2^ Developments in DNA synthesis (writing), including enzymatic DNA synthesis,^3^ and next-generation sequencing (reading) technologies such as Illumina Sequencing and nanopore sequencing (Oxford Nanopore Technologies),^4–6^ have brought DNA data storage from a purely scientific exercise very close to a realistic and attractive archival medium.

However, the integrity of the stored data and its retrieval from DNA-based memory systems pose some challenges. To ensure durability and data preservation, many methods have been explored to protect DNA molecules against environmental degradation that could impair data fidelity over time. New nanomaterials have been investigated to ensure the longevity of DNA fragments for storage, such as silica nanoparticles,^7^ reusable and non-destructive electrospun nanofiber matrices,^8,9^ thermoresponsive microcapsules^10^ and metal–organic frameworks (MOFs).^11^ Moreover, robust error-correcting codes have been developed to ensure high-fidelity retrieval, such as the Goldman method,^12^ DNA Fountain^13^ and the Yin-Yang Codec.^14^

Besides integrity, an essential requirement for scaling up DNA data storage systems is random access: the capacity to selectively retrieve isolated and specific data sets without having to read out the entire archive. To date, this is most often accomplished by polymerase chain reaction (PCR) amplification, which relies on the use of primers to locate and amplify specific DNA sequences.^15^ While extensively utilized, random access based on PCR suffers from a variety of drawbacks: it is error-prone through misincorporation of nucleotides by the polymerase, sensitive to primer-template mismatches and can generate biases in amplification.^16^ Such issues can lead to the corruption of data and loss of non-target sequences, especially if competing sequences are present in the same reaction, impacting the overall retrievability of the stored information. Moreover, the process is energy-demanding, requiring thermal cycling, specialized reagents, and optimized specific conditions that also increase time and cost.

Several PCR-independent random access methods exist, including the use of photonic microspheres with optical microscopy,^17^ multicolor fluorescently-tagged microparticles and flow cytometry,^18^ and Cas9-directed selection mechanisms.^19^ However, these techniques often require specialized, expensive equipment, and complex procedures, making these methods more difficult for end-users.

Here we present a proof of concept of a facile PCR-independent selection approach for encoded DNA, also serving as a double-layered protection system for data storage and DNA preservation. The process eliminates the need for selective enzymatic amplification and can carry out targeted retrieval of isolated sequences co-located in the same physical space. By encapsulating data-encoded DNA sequences inside the lipidic spherical bilayer of stimulus-sensitive small unilamellar vesicles (SUVs), we provide a system where the release of information is selectively triggered by external stimuli. Our technique offers a sequential and selective data recovery system without disrupting non-targeted data.

Stimuli-responsive vesicle systems have been used in drug delivery to induce targeted cargo release by external stimuli such as pH change, enzymatic catalysis, and interaction with the cytoplasm.^20–22^ Here, we apply this concept to digital data storage by encapsulating synthetic DNA sequences in distinct vesicle populations. We report the initial steps of an addressable storage system incorporating pH-responsive SUVs that exhibit orthogonal responses under acidic and alkaline conditions.

To obtain SUVs sensitive to acidic pH values, we drew on vesicles composed of the zwitterionic lipid 1,2-di-(9*Z*-octadecenoyl)-*sn*-glycero-3-phosphoethanolamine (DOPE) and the anionic lipid 3β-hydroxy-5-cholestene 3-hemisuccinate (CHEMS), which are known to release cargo upon protonation.^20–22^ Unlike acidic pH-responsive SUVs, basic pH-responsive SUVs have not been widely reported. We chose anionic 1,2-dioctadecanoyl-*sn*-glycero-3-phosphate (DSPA) and zwitterionic 1,2-dihexadecanoyl-*sn*-glycero-3-phosphocholine (DPPC) lipids to provide sensitivity to basic pH values.

The vesicles can further be stabilized and protected by electrospun polymer fibers.^23^ Electrospinning is an electrohydrodynamic process whereby nanofibrous meshes are generated from a jet of polymer solution exposed to electrical forces.^24^ By adding SUVs to the polymer solution, we embed them within the fibers during the process. From this system, which is straightforward to produce and handle, we demonstrate the retrieval of specific DNA-encoded data sets from water-resistant polymeric nanofibers (Fig. 1).

**Fig. 1:**
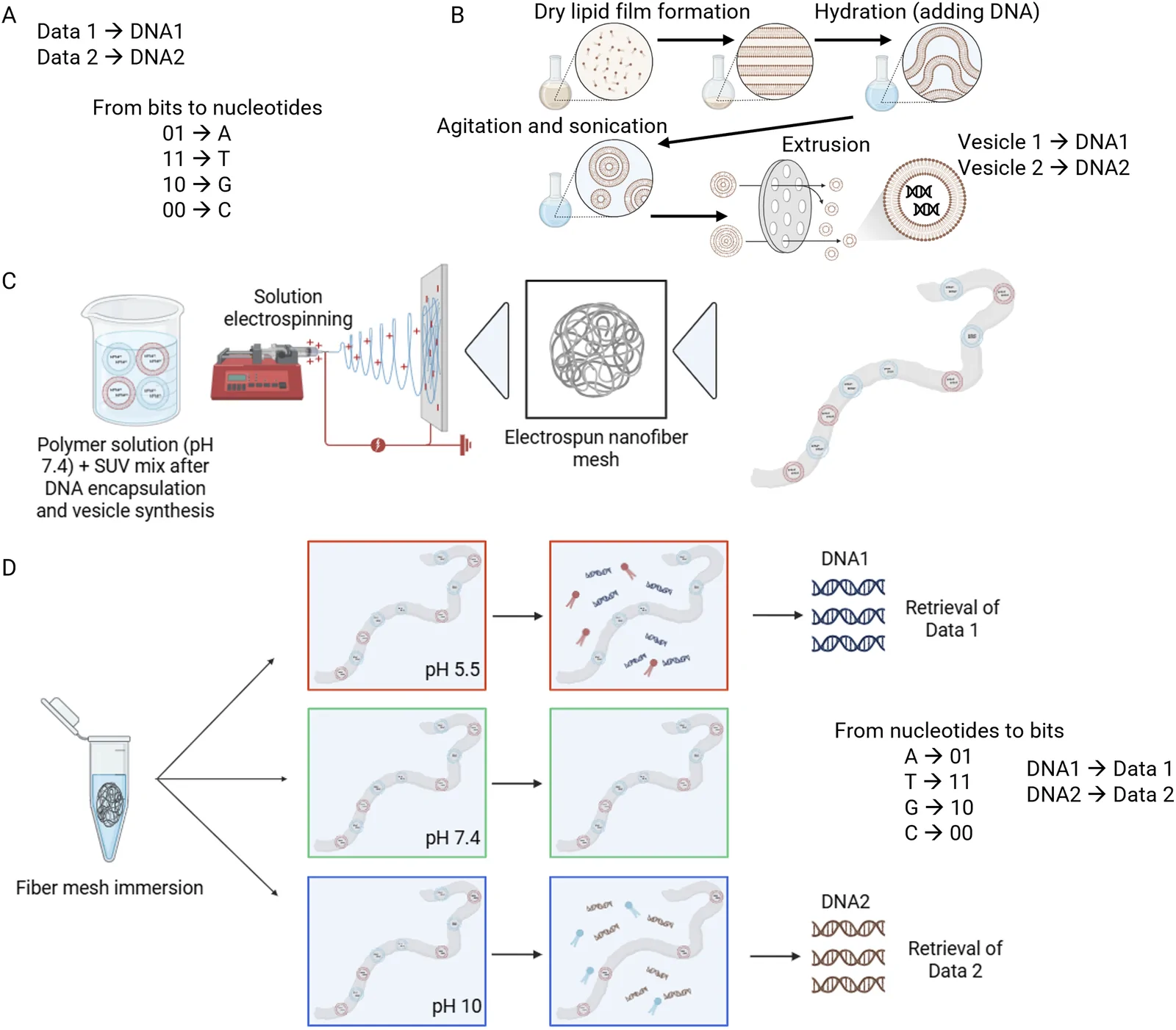
Overview of the PCR-independent random access experimental workflow. (A) Data is converted to DNA sequences using a simple encoding scheme. (B) Synthesis of specific DNA-loaded SUVs by dry lipid film formation and extrusion. Each SUV type encapsulates a specific DNA sequence. (C) Both orthogonally responsive SUVs are mixed in a polymer solution at physiological pH. This mix is then electrospun to obtain the nanofiber mesh and later exposed to UV for cross-linking. A water-resistant fiber mesh is obtained. (D) Random access and sequential retrieval experiments are performed using different pH fiber immersions. Targeted data is retrieved and decoded.

## Results and discussion

### Design, synthesis, characterization and response mechanism of SUVs

All SUVs were synthesized using thin-lipid film hydration followed by extrusion (Materials & Methods). They were characterized by dynamic light scattering (DLS).

First, as a control of the SUV synthesis protocol, neutral DMPC : DPPC (1,2-ditetradecanoyl-*sn*-glycero-3-phosphocholine, 1,2-dihexadecanoyl-*sn*-glycero-3-phosphocholine) SUVs were produced. DLS measurements (Fig. S1 and S2) showed a mean diameter of 102.4 ± 6.8 nm with a polydispersity index (PDI) of 0.042, consistent with a highly monodisperse vesicle solution. These results validate the selection of thin-lipid film hydration and extrusion for SUV synthesis procedure.

The same process was repeated for empty pH-responsive SUVs. The stability of the empty vesicles in solution was evaluated under different pH conditions (Fig. 2A bright red, Fig. 2B bright blue). In the case of DOPE : CHEMS, under acidic conditions the size increased approximately tenfold (1669.6 ± 254.7 nm, Fig. S3A), while at physiological and basic pH the vesicles maintained constant diameters of 121.6 ± 9.2 nm and 127.4 ± 4.7 nm, respectively (Fig. S3B and S3C). This reflects the conformational change of DOPE into an inverted hexagonal phase and aggregation-prone structure when CHEMS is protonated^25,26^ at pH < 6. This increase in size can be attributed to disruption, lipid fusion, and aggregation with neighboring SUVs, as large sediments were formed after 24 h at pH 5.5. Under this condition, the sample exhibited extreme aggregation (PDI = 1.000), and the cumulants-derived Z-average (∼14.5 µm) was not representative of particle size (which differed from intensity mean values).

**Fig. 2:**
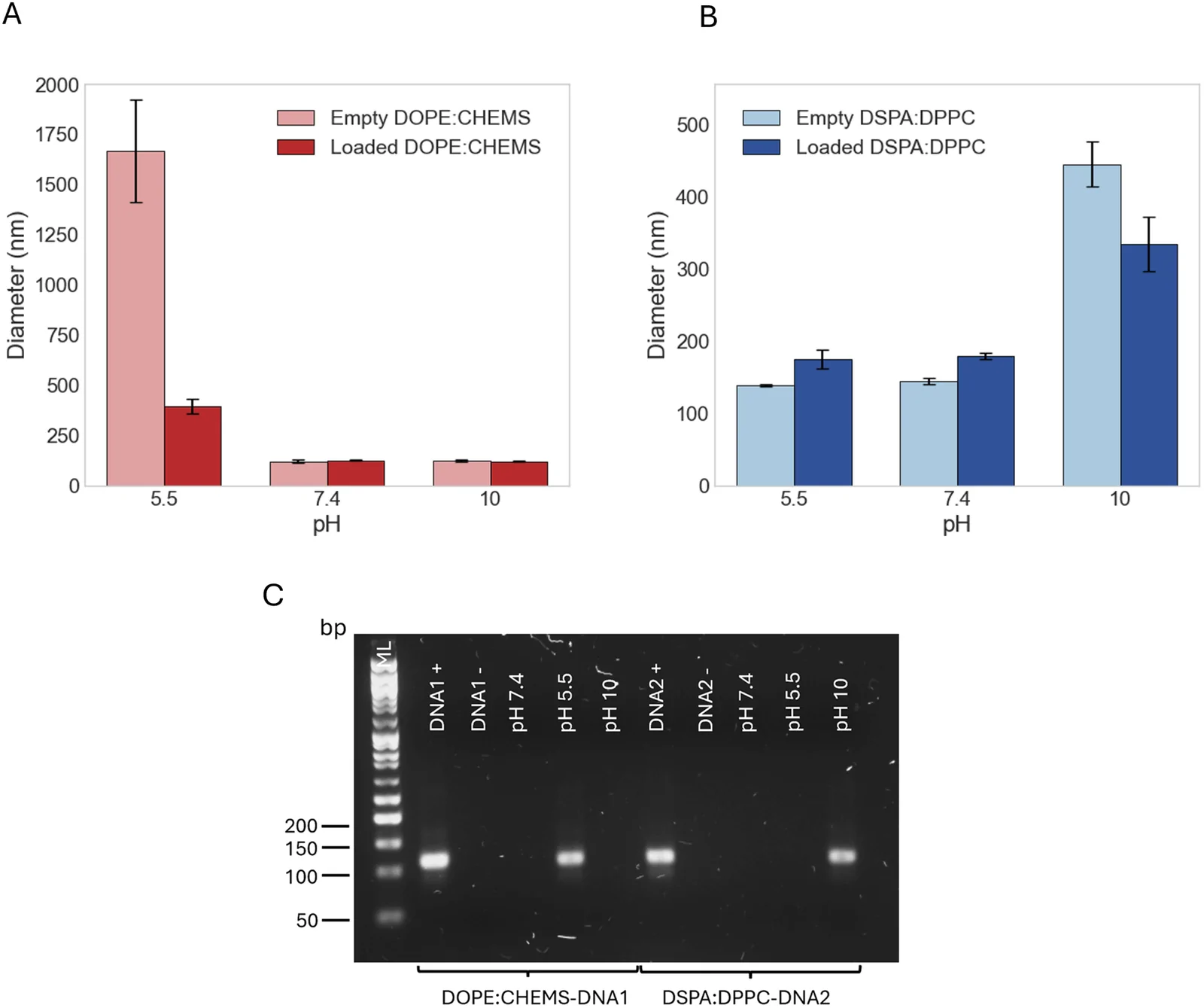
DLS results showing the specific triggered response of synthesized empty and loaded pH-responsive SUVs, and targeted in-solution DNA release. (A) Diameter size distribution of DOPE : CHEMS vesicles before and after DNA encapsulation, measured by DLS at *t* = 0 under different pH conditions. (B) Diameter size distribution of DSPA : DPPC vesicles before and after DNA encapsulation, measured by DLS at *t* = 0 under different pH conditions. (C) Agarose gel electrophoresis of DNA release from pH-responsive lipid vesicles in solution, after PCR amplification. Lanes from left to right: DNA marker ladder (50 bp); DNA1 positive control with PS1; DNA1 negative control; sample from DOPE : CHEMS pH 7.4 solution with PS1; sample from DOPE : CHEMS pH 5.5 solution with PS1; sample from DOPE : CHEMS pH 10 solution with PS1; DNA2 positive control with PS2; DNA2 negative control; sample from DSPA : DPPC pH 7.4 solution with PS2; sample from DSPA : DPPC pH 5.5 solution with PS2; sample from DSPA : DPPC pH 10 solution with PS2.

In the case of the DSPA : DPPC vesicles, the diameter (Fig. 2B, bright blue) remained stable under both acidic and physiological pH conditions (144.9 ± 4.8 nm and 139.5 ± 1.6 nm, respectively; Fig. S4A and S4B), while it tripled when exposed to alkaline conditions (445.6 ± 31.3 nm, Fig. S4C). This is consistent with DSPA becoming doubly deprotonated when exposed to alkaline pH,^27^ causing an increase in the repulsive charges on the polar head groups on the external side of the membrane, which destabilizes the vesicles, producing a gradual increase in diameter. Such membrane destabilization should promote partial or full release of encapsulated material.

### DNA encapsulation and release from SUVs in solution

Next, to test the targeted release of DNA from each pH-responsive SUV, we encoded two different messages in two different DNA oligonucleotides. Each message was encoded using a binary conversion scheme (A: 01, T: 11, G: 10, C: 00) and repeated twice to introduce redundancy and preserve information integrity. The resulting single-stranded DNA (ssDNA) strands were flanked with primer binding regions for a total of 112 bases and synthesized, with DNA1 encoding the word “Nanogune” and DNA2 encoding the word “Textadna” (Fig. S5).

We used these primer binding sites (Fig. S5) to design and synthesize forward and reverse primers, for the DNA release verification steps. Primer set 1 (PS1) amplifies DNA1, and primer set 2 (PS2) amplifies DNA2 (Table S1). PCR was introduced as a readout step to confirm successful DNA release and to verify the orthogonality of the system, when both the primer set and its matching DNA are present.

To evaluate DNA release from the pH-responsive SUVs, DNA1 was encapsulated in DOPE : CHEMS vesicles and DNA2 in DSPA : DPPC, with encapsulation efficiencies of 91.4% and 95.7%, respectively (Formula S1; Materials & Methods). The vesicles were then subjected to pH changes, and their hydrodynamic diameters were measured by DLS. As observed in Fig. 2A (dark red) and Fig. 2B (dark blue), all samples followed size trends comparable to those of the previously characterized empty SUV samples.

In the case of DNA1-loaded DOPE : CHEMS vesicles, the sample at pH 5.5 reached a diameter of 397.8 ± 36.1 nm. For DNA2-loaded DSPA : DPPC vesicles, exposure to pH 10 triggered a doubling of their diameter (335.3 ± 37.8 nm). In both cases, under the triggering conditions, the loaded SUVs showed a smaller diameter increase at *t* = 0 than the empty vesicles.

To confirm that the conformational changes observed in the SUVs under different pH conditions resulted in selective DNA release (Fig. S6), we collected the supernatants of the different samples and we subjected them to PCR and gel electrophoresis analysis. In Fig. 2C, DNA amplification was observed under acidic conditions for DNA1 encapsulated in DOPE : CHEMS, and under alkaline conditions for DNA2 encapsulated in DSPA : DPPC vesicles. These results confirm DNA retrieval under targeted pH conditions.

### Encapsulation of SUVs in electrospun nanofibers

To advance towards dry physical matrices capable of storing multiple data sets, we embedded the DNA-encapsulated SUVs in electrospun nanofiber matrices. These matrices constitute a stable platform for storing DNA and provide a dry storage system with limited energy usage.^8,9^

Based on our previous studies,^9^ a water-resistant polymer formulation was identified and selected as the embedding matrix for this work. Specifically, due to chemical and solvent compatibility with the SUVs, we employed nanofibers composed of polyethylene oxide : trimethylolpropane triacrylate (PEO : TMPTA; cPEO), cross-linked in the presence of benzophenone under 254 nm UV illumination, to encapsulate different SUVs (Materials & Methods). Under UV light, benzophenone abstracts hydrogen from PEO, generating radicals that initiate free-radical polymerization of TMPTA groups and form a cross-linked polymer network.

To assess the stability of the SUVs before and after cross-linking, arbitrarily selected empty DSPA : DPPC SUVs were tested. First, to check the stability in solution, the sample was exposed to UV at a 254 nm wavelength, the same condition used for cross-linking. Based on DLS measurements, a 2 h UV exposure maintained SUV stability in solution (Table S2). This exposure time was sufficient to render the fibers water-resistant post-electrospinning, while maintaining SUV stability both in solution and when embedded in fibers. This conclusion is supported by subsequent retrieval experiments, which showed no significant DNA release after the first immersion of the fibers.

As a control experiment to assess SUV distribution within the nanofibers, we added the lipophilic fluorescent dye DiO to neutral SUVs (DMPC : DPPC) during the synthesis process, at the lipid dissolution step. For this visualization-only experiment, PEO was preferred due to its hydrophilicity and facile dissolution and fiber production. After electrospinning a 10% w/w PEO polymer solution containing 10% v/v (*ca.*) labeled SUV solution (Materials & Methods), the resulting dyed vesicle-containing nanofibers were observed by fluorescence microscopy (Fig. 3).

**Fig. 3:**
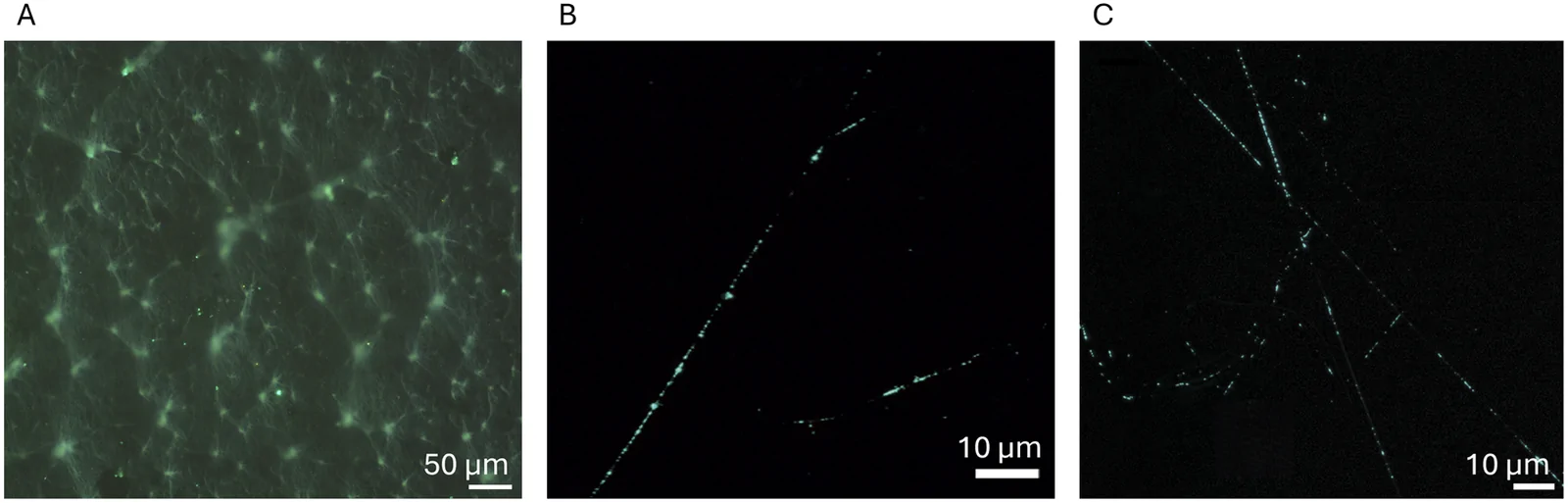
Fluorescence microscopy images of fluorescently labeled SUVs embedded in a thin electrospun nanofiber mesh (A) and in individual nanofibers (B and C). DiO in the SUV membrane emits at 506 nm (green) when excited at 488 nm (blue). Scale bars: 50 µm (A) and 10 µm (B and C).

Fig. 3A shows the fluorescence signal detected after the incorporation of fluorescently labeled SUVs into a thin electrospun fiber mesh. Observation of the same region at lower magnification (Fig. S7) revealed a relatively homogeneous fluorescence distribution across the mesh, suggesting uniform vesicle incorporation over a larger area. At higher magnification, the histogram and the three-dimensional fluorescence intensity profiling of this region (Fig. S8) further demonstrated a heterogeneous yet spatially well-distributed fluorescence pattern due to random fiber accumulation areas. The broad distribution of fluorescence peaks indicates successful incorporation and spatial dispersion of lipid vesicles within the electrospun matrix, with no significant evidence of macroscopic aggregation.

These fluorescently labeled vesicles exhibited a discrete heterogeneous distribution along individual fibers at the microscopic scale (Fig. 3B and C). However, as previously mentioned, at the macroscopic scale this heterogeneity becomes insignificant. As we demonstrate later in this work, despite the heterogeneous vesicle distribution at the individual-fiber level, selective retrieval is consistently achieved from macroscopic-scale fiber sections, indicating that the functionality of the electrospun mesh is preserved regardless of the local vesicle distribution pattern.

Regarding fiber size, the mean nanofiber diameter was 387.9 ± 82.7 nm, as measured from SEM images using ImageJ. Non-dense or underpopulated areas of nanofibers are shown in the SEM images in Fig. 4.

**Fig. 4:**
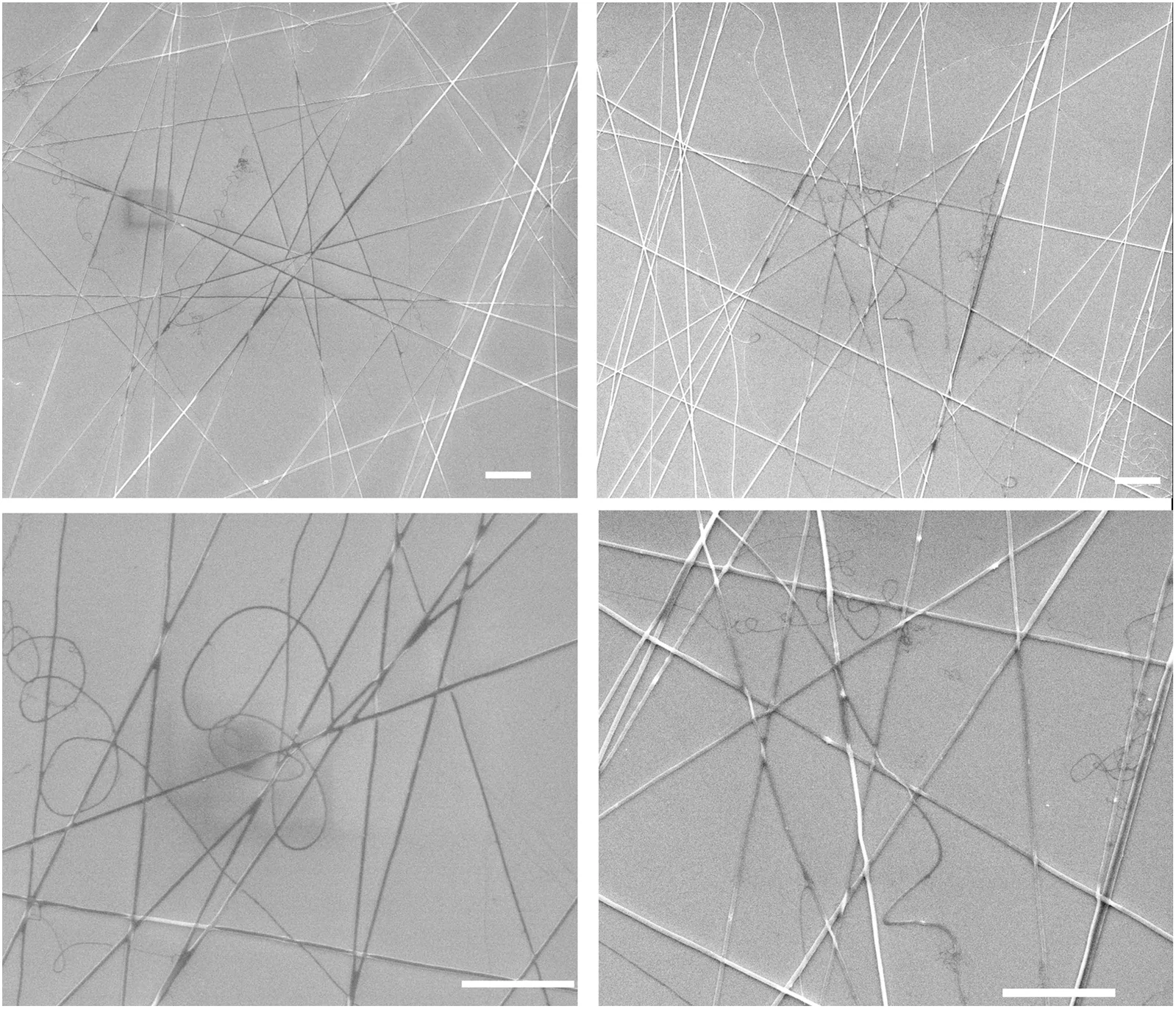
SEM images of electrospun SUV-PEO nanofibers showing non-dense or underpopulated nanofiber areas. Scale bar: 10 µm.

### DNA release from SUVs embedded in polymeric fiber

Following the incorporation of SUVs into polymeric nanofibers, we investigated DNA release from the resulting fiber matrices. First, DNA release from neutral SUVs embedded in fibers was tested against the release from fibers with directly embedded DNA to assess the benefits of double-layered encapsulation. For this experiment, non-cross-linked PEO was selected as the polymer matrix. Electrospun nanofibers were prepared from polymer solutions of 10% w/w PEO with 10% v/v (*ca.*) DNA1-loaded SUVs (DMPC : DPPC) and 10% w/w PEO containing DNA1 at a final concentration of 0.0015% w/w (Materials & Methods).

We used agarose gel band intensities for relative quantification of DNA release from both sets of fibers. In the case of PEO-DNA1 fibers, normalization was performed by setting the 1 h incubation at 80 °C as the 100% release value, based on the complete dissolution of the fiber mesh under these conditions.^8,9^

Fig. 5 shows that within 5 min of immersion of PEO-DNA1 fibers, 67.7% of the DNA was already released. The release reached 98.7% after 24 h at room temperature (RT) and after 1 h at 37 °C.

**Fig. 5:**
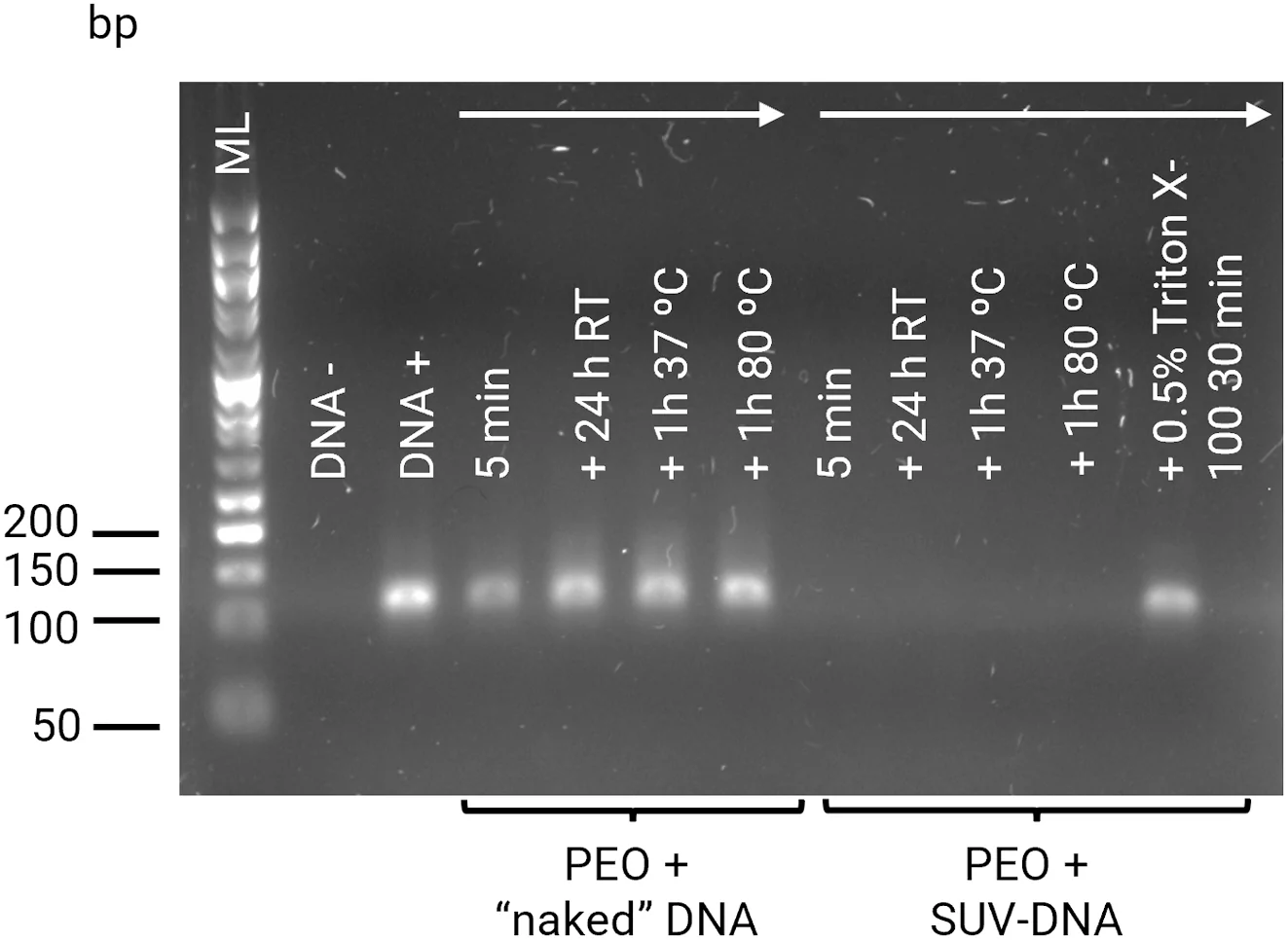
Agarose gel electrophoresis (2%) results for DNA release from “naked” DNA – PEO fibers *vs.* encapsulated DNA in SUVs – PEO fibers. Normalized band intensities are used to calculate the relative release. The arrows indicate the consecutively applied conditions, from which equal volume samples were taken.

On the other hand, the fibers in which the DNA-containing SUVs were embedded exhibited no measurable DNA release after 24 h of incubation at RT, or after heating the samples to 80 °C (the SUV-fiber matrix was not fully dissolved under these conditions). This result suggests that, even when the system reaches the melting point of these SUVs (34 °C), the permeability is not sufficient for the DNA to diffuse out of the vesicles. Finally, DNA release was detected after adding 0.5% Triton X-100, which causes complete disruption of the SUVs.

These results show that the SUVs provide a second layer of protection for the DNA and enable on-demand release when embedded in nanofiber matrices. Moreover, a minimal amount of surfactant (Materials & Methods) was sufficient for significant DNA recovery, further supporting on-demand DNA release from the vesicles.

Going further towards an orthogonal stimuli-responsive and water-resistant system, and to evaluate the behavior of the SUVs once embedded in the fibers, we synthesized two independent cPEO fiber meshes (Materials & Methods), each embedding either DNA1-loaded DOPE : CHEMS or DNA2-loaded DSPA : DPPC. Each was subjected to fiber immersion cycle – DNA release experiments.

Fig. 6 shows the DNA retrieval from each independent fiber mesh verified by PCR amplification and gel electrophoresis. Equally sized cuts from each nanofiber mesh were immersed in different consecutive pH solutions, up to values at which the relevant SUVs are disrupted. In the first fiber mesh (Fig. 6, left), DNA was amplified and detected on the agarose gel only when the mesh was immersed in pH 5.5 acetate buffer solution. In the second fiber mesh (Fig. 6, right), DNA was only present in the sample where the mesh was immersed in pH 10 NaOH solution. Both SUVs displayed the same behavior in solution and when embedded in the fibers.

**Fig. 6:**
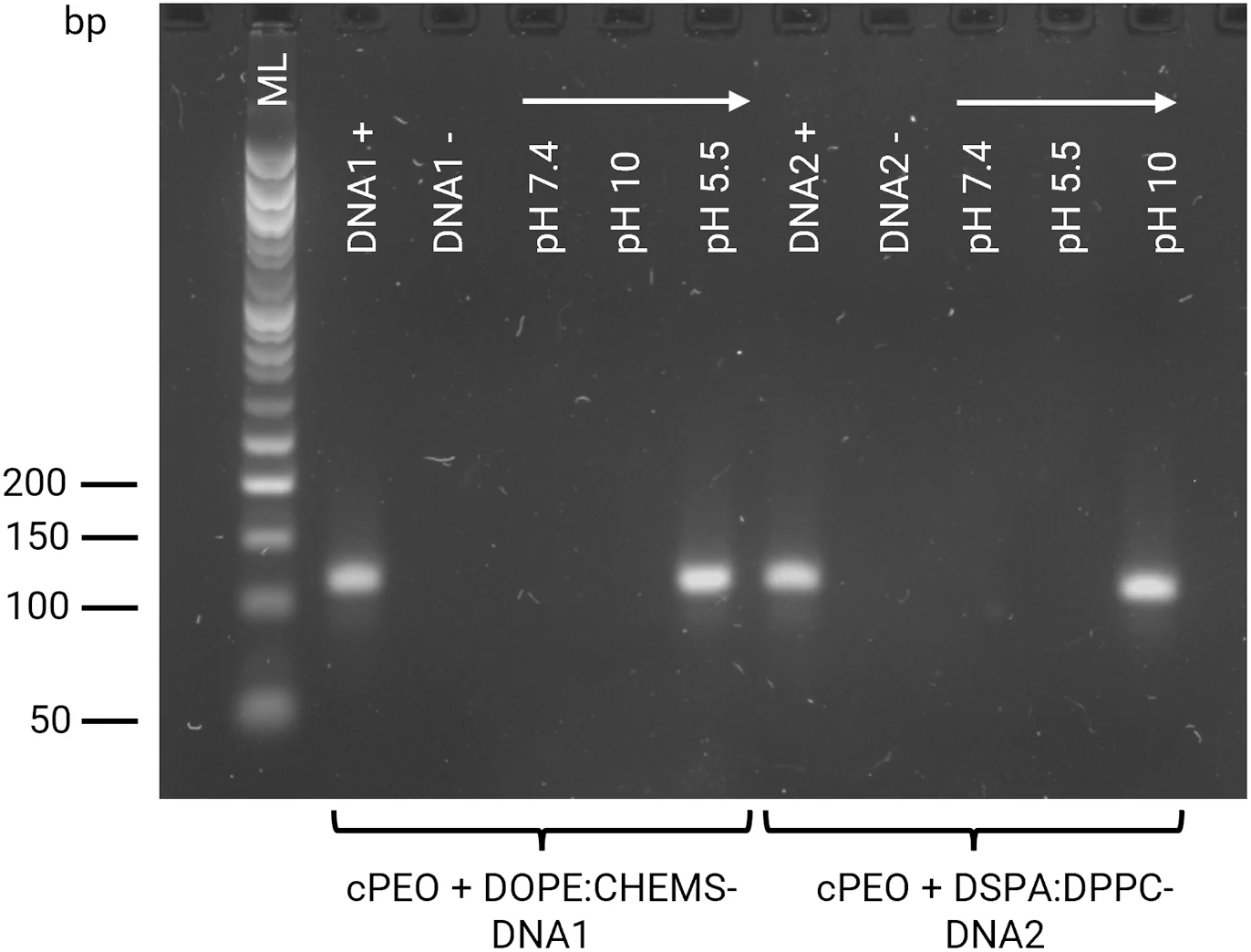
DNA release from independent fiber meshes shown in 2% agarose gel electrophoresis, after PCR amplification. On the left, the DNA marker ladder. Next, the DNA release experiment on the first fiber mesh (cPEO + DOPE : CHEMS-DNA1). Lastly, the DNA release experiment on the second fiber mesh (cPEO + DSPA : DPPC-DNA2). The arrows indicate the order of the immersions, with the DNA released in the final immersion. From left to right: DNA marker ladder (50 bp); DNA1 positive control with PS1; DNA1 negative control; sample from cPEO + DOPE : CHEMS immersion in pH 7.4 solution with PS1; sample from cPEO + DOPE : CHEMS immersion in pH 10 solution with PS1; sample from cPEO + DOPE : CHEMS immersion in pH 5.5 solution with PS1; DNA2 positive control with PS2; DNA2 negative control; sample from cPEO + DSPA : DPPC immersion in pH 7.4 solution with PS2; sample from cPEO + DSPA : DPPC immersion in pH 5.5 solution with PS2; sample from cPEO + DSPA : DPPC immersion in pH 10 solution with PS2.

For both meshes, no amplifiable DNA was detected after the first and second solution immersions. This further demonstrates that the UV-cross-linked meshes preserved vesicle functionality, limited substantial premature leakage during the initial immersion cycles, and retained the pH-selective release feature of the vesicles.

### Use of orthogonal trigger-responsive SUVs to achieve molecular random access

To demonstrate molecular random access using orthogonal stimuli, we encapsulated both independently trigger-responsive SUV populations in a single cPEO nanofiber matrix system. Prior characterization confirmed that each SUV-DNA system responded selectively to its intended stimulus (see above). The experimental workflow for this random access DNA retrieval is described in Materials & Methods (Fig. 1), which follows the general scheme of the previous experiments. PCR amplification followed by agarose gel electrophoresis was used as an initial verification method for these random access experiments.

As an initial control, PCR-free random access experiments were performed in solution by mixing both SUV populations under identical conditions. In Fig. S9, DNA release was observed only under the correct combination of conditions: when PS1 was added to the solution extracted from the pH 5.5 condition, and when only PS2 was added after sample extraction from pH 10 solution.

These results suggest that when both SUV populations are mixed, the vesicles remain intact and that there is no significant interaction (aggregation or fusion) between them. More importantly, they follow the same DNA release pattern shown in “independent” solution release experiments, demonstrating that the selective access of targeted DNA is feasible in solution. However, because long-term SUV stability in solution is limited due to aggregation, solid-state storage within electrospun fibers was pursued as the preferred matrix.

Moving on to the dry system, after introducing both SUVs in the same polymeric mixture, we obtained a single cPEO mesh. Random access and fiber sequential retrieval experiments were conducted as explained in Materials & Methods, which consisted of two different experiments following distinct fiber mesh immersion cycles. The experiments were performed using a single time-point measurement (30 min) for a binary (triggered/not triggered) readout of the release.

The first experiment (immersion cycle: pH 7.4 → 5.5 → 10, Fig. 7A and B) shows that no DNA amplification was detected after immersion in pH 7.4 1× PBS solution, indicating no DNA release, thus supporting SUV integrity after electrospinning. Upon immersion in pH 5.5 solution, only the sample subjected to PS1 was amplified, demonstrating selective release of DNA1 and confirming that only DOPE : CHEMS vesicles were triggered under acidic conditions.

**Fig. 7:**
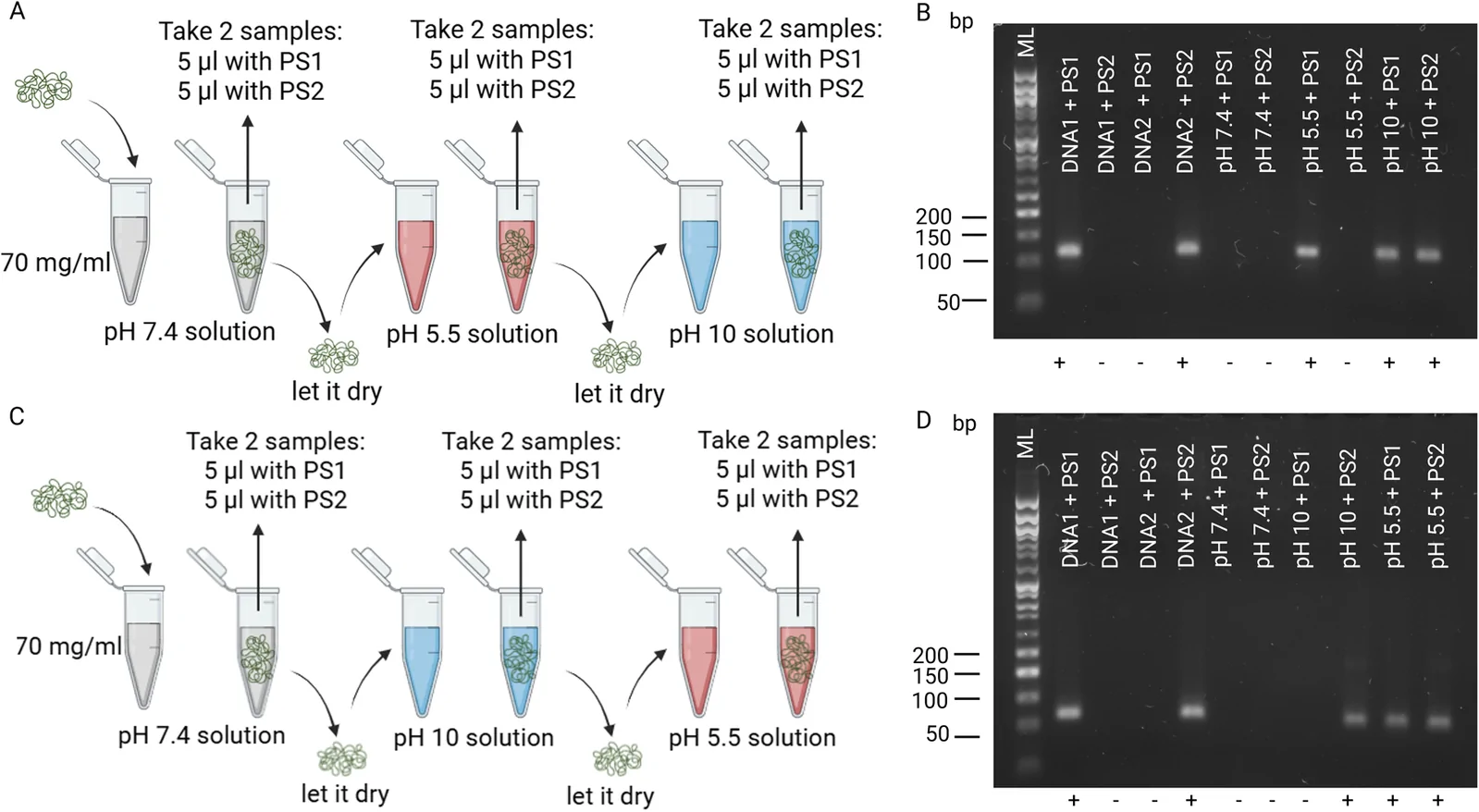
Schematic representation and agarose gel electrophoresis results of the first (A and B) and second (C and D) proposed experiments for random access. (A) Fiber mesh immersion cycles (pH 7.4 → 5.5 → 10) and sample harvesting for future verification-PCR. (B) 2% agarose gel electrophoresis after verification-PCR. From left to right: DNA marker ladder (50 bp); DNA1 positive control with PS1, DNA1 negative control with PS2; DNA2 negative control with PS1; DNA2 positive control with PS2; sample from pH 7.4 immersion with PS1; sample from pH 7.4 immersion with PS2; sample from pH 5.5 immersion with PS1; sample from pH 5.5 immersion with PS2; sample from pH 10 immersion with PS1; sample from pH 10 immersion with PS2. The lower bar: + DNA presence (after PCR); – DNA absence (after PCR). (C) Fiber mesh immersion cycles (pH 7.4 → 10 → 5.5) and sample harvesting for future verification-PCR. (D) 2% Agarose gel electrophoresis after verification-PCR. From left to right: DNA marker ladder (50 bp); DNA1 positive control with PS1; DNA1 negative control with PS2; DNA2 negative control with PS1; DNA2 positive control with PS2; sample from pH 7.4 immersion with PS1; sample from pH 7.4 immersion with PS2; sample from pH 10 immersion with PS1; sample from pH 10 immersion with PS2; sample from pH 5.5 immersion with PS1; sample from pH 5.5 immersion with PS2. Lower bar: + DNA presence (after PCR); – DNA absence (after PCR).

To retrieve the second data set, the same fiber mesh was recovered, dried and immersed in the pH 10 solution. As shown by electrophoresis (Fig. 7B), now DNA amplification was detected with both primer sets. The DNA signal with PS2 corresponded to DNA2 release, confirming the triggering of DSPA : DPPC vesicles under alkaline conditions. The DNA signal with PS1 would be attributed to the free DNA1 remaining within the fiber mesh following the initial acidic trigger. This process was repeated, introducing an additional washing step (1× PBS wash and drying). This step eliminated the residual carried-over DNA1 and made the fiber system suitable for a clean subsequent retrieval.

To further validate the orthogonality, a second experiment was performed by swapping the order of the alkaline and acidic pH solutions (immersion cycle: pH 7.4 → 10 → 5.5) as documented in Fig. 7C and D. Consistent with the first experiment, no DNA release was observed at pH 7.4, again confirming SUV stability after electrospinning.

When fibers were first immersed at pH 10, amplification occurred only with PS2, indicating selective release of DNA2 from DSPA : DPPC vesicles. The same fiber mesh was used to retrieve the other data set. Following drying and re-immersion at pH 5.5, amplification was detected with both primer sets. The DNA signal with PS2 was attributed to residual free DNA2 from the prior alkaline trigger, and a washing step was added to remove it. The DNA signal after subjecting to PS1 matched the DNA1 release, confirming the selective triggering of DOPE : CHEMS vesicles under acidic conditions.

To compare the released DNA fractions and independently assess pH-specific DNA release, qPCR experiments were performed (Materials & Methods). The released DNA values were derived from the quantification cycle (*C*_q_) measurements (Fig. S10) using the standard curve generated for each primer set (Fig. S11).

In the case of the samples subjected to PS1, the no-template control (NTC) sample and the samples immersed at pH 7.4 and pH 10 showed a very similar *C*_q_ (*C*_q_ ≈ 28), indistinguishable from the NTC and therefore treated as no specific release. The samples at pH 5.5 showed true amplification with a low *C*_q_ value (∼15), well below the NTC threshold. Since *C*_q_ scales inversely with the logarithm of the released DNA amount (lower *C*_q_ = more DNA), this indicates that DNA1 was specifically retrieved under the pH 5.5 trigger in both experiments.

On the other hand, in the case of the samples subjected to PS2, similarly, the NTC sample as well as the samples immersed at pH 5.5 and pH 7.4 showed a very similar *C*_q_ (*C*_q_ ≈ 26), indistinguishable from the NTC and therefore considered as showing no specific release. The samples at pH 10 showed significant amplification with a low *C*_q_ value (∼12). DNA2 was specifically retrieved under the pH 10 trigger in both experiments.

In the first experiment, no leaked DNA was measured at the first pH 7.4 immersion. A total of 67.43 ± 41.42 pg DNA1 per mg fiber was released at pH 5.5 immersion, with no significant leakage from non-targeted SUVs. After the washing cycle, 34.04 ± 6.97 pg DNA2 per mg fiber was measured at pH 10 immersion, with no residual DNA1 carried over from the previous triggered release.

The second experiment gave the same result after the initial pH 7.4 immersion. A total of 25.85 ± 9.14 pg DNA2 per mg fiber was measured after the pH 10 immersion. Finally, after washing and drying the same fiber mesh, 26.48 ± 11.72 pg DNA1 per mg fiber was released after the pH 5.5 solution immersion. The washing step removed the residual DNA2 fraction from the dried samples.

In these experiments, for both primer sets the NTC signals appeared after 26 cycles, more than 5 cycles after the lowest standard concentration (1 pM), which is acceptable. Based on Formula S2, for PS1 the NTC's background signal was around 1.3%, while for PS2 it was around 1.1%, much lower than the maximum acceptable limit of 3%, emphasizing the specificity of the experiment and the reliability of the results.

Together, these two orthogonal experiments demonstrate pH-addressable control over cargo release within a single fiber matrix: at pH 5.5 only DNA1 is released, as only DOPE : CHEMS vesicles are triggered, while at pH 10 only DNA2 is retrieved, as only DSPA : DPPC vesicles are triggered. Non-targeted DNA remained intact in the fiber mesh for further retrieval purposes. These results establish pH-triggered SUV activation as an effective and fast strategy for programmable, selective DNA release from co-localized vesicle populations, embedded within the same material. It is worth mentioning that only a cut of the fiber mesh is used during a given retrieval process (one full immersion cycle), while the rest of the mesh still retains the encapsulated DNA/data and can still be accessed subsequently.

To prove the selectivity of the system, Sanger sequencing experiments were performed on the amplified samples, and on DNA1 and DNA2 as synthesized for positive controls.

The first sequence iteration showed reduced base-calling quality near the 5′ end; consequently, only the second iteration was reliably recovered by Sanger sequencing. Noise near the primer-binding region may account for this. After analyzing the sequenced samples, Fig. 8 shows a precise alignment in the second iteration of the message between the DNA released at pH 5.5 and DNA1, and between the DNA released at pH 10 and DNA2. No other oligonucleotide fragments were detected in either sample. These results confirm that pH 5.5 specifically triggered DOPE : CHEMS vesicles, which were initially loaded with DNA1, and that pH 10 only triggered DSPA : DPPC vesicles, which were loaded with DNA2. The correct retrieval and decoding of the original messages were also possible due to the strand architecture of the oligonucleotides, since a successful sequencing of one of the two iterations was sufficient.

**Fig. 8:**
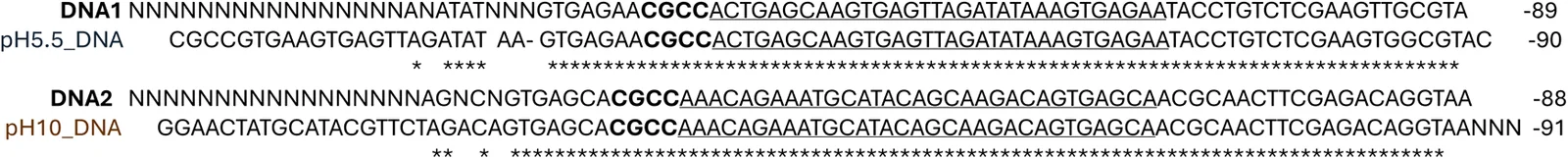
Processed Sanger sequencing results, after base-calling of the raw data. With the stars, the bases that are overlapped with their respective control samples; underlined, the payload of the encoded data (second iteration); in bold, the “CGCC” spacers.

### 24-Hour high-temperature stress test for system stability evaluation

We performed a short high-temperature stress test to assess the stability of the system under specific extreme environmental conditions. The samples were exposed to a relative humidity of 50% for 24 h, at 85 °C (Materials & Methods). DNA release experiments were performed, using the same methodology used for random access experiments (random access experiment 2 was arbitrarily chosen: pH 7.4 → 10 → 5.5).

From the agarose gel electrophoresis results of the samples after PCR (Fig. 9), no DNA signal was detected when submerging the stressed samples in 1× pH 7.4 PBS solution, after exposing them to both primer sets (PS1 and PS2). This suggests that both SUVs remains intact, trapped in nanofiber meshes and offering strong DNA protection.

**Fig. 9:**
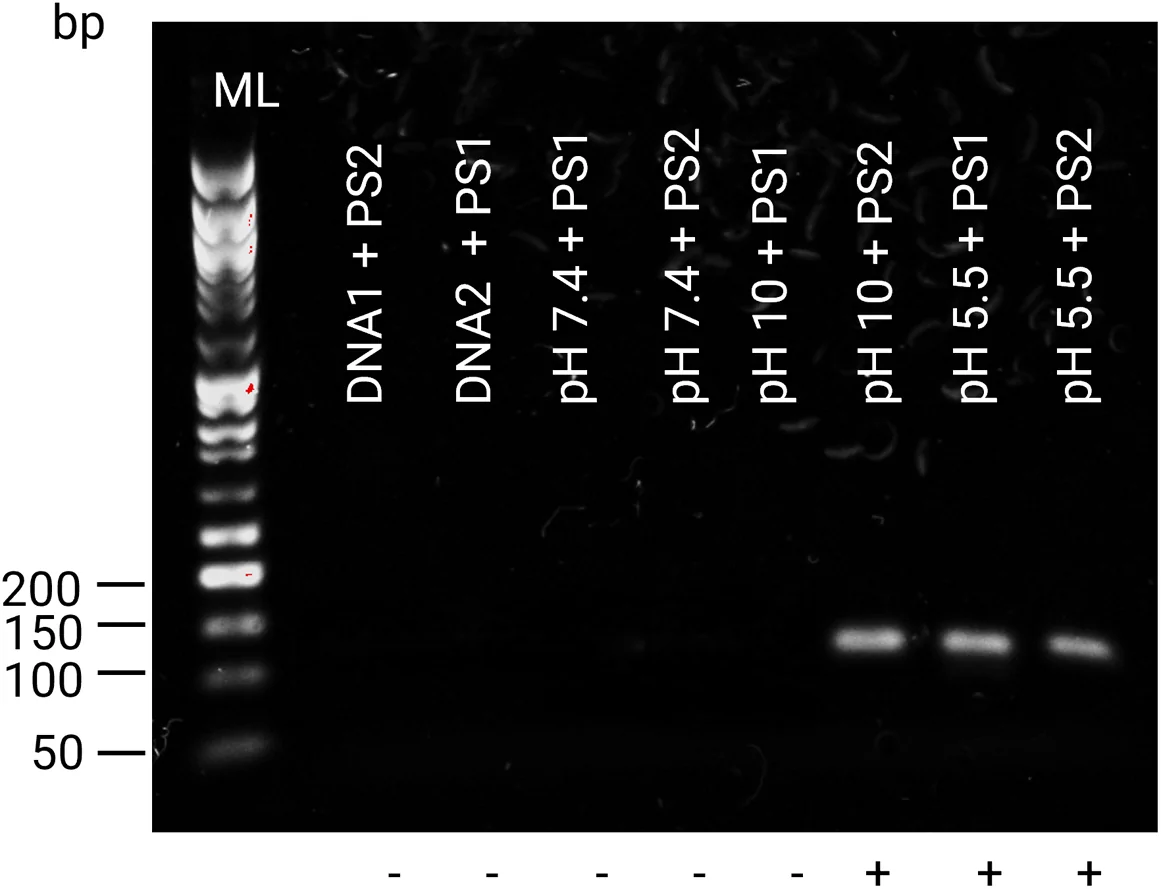
Agarose gel electrophoresis results from the second random access experiment, after subjecting the fiber mesh to specific stress conditions. From left to right: DNA marker ladder (50 bp); DNA1 negative control with PS2; DNA2 negative control with PS1; sample from pH 7.4 immersion with PS1; sample from pH 7.4 immersion with PS2; sample from pH 10 immersion with PS1; sample from pH 10 immersion with PS2; sample from pH 5.5 immersion with PS1; sample from pH 5.5 immersion with PS2. The lower bar: + DNA presence (after PCR); – DNA absence (after PCR).

Finally, upon exposure to changes in pH conditions, the SUVs responded in the same way as observed in the non-stressed random access experiments. These results support the conclusion that this selectivity remains intact after exposure to specific stress conditions, with the non-targeted DNA still being protected. These findings suggest that the system could be suitable for longer-term storage and retrieval, and motivate future work in this direction.

## Conclusions

This work provides a proof of concept for a PCR-independent selection mechanism for molecular random access in DNA data-storing systems. By combining nanofiber matrices with encapsulated SUVs responsive to stimuli, we present a retrieval mechanism for targeting DNA sequences *via* orthogonal external triggers (pH) while keeping the rest of the information intact within the system. With this system we have laid the first foundations for a physical confinement of individual DNA-data files providing a double layer of protection for data storage and integrity, sequence-independent pH-triggered selection, and preservation of non-triggered compartments during targeted access.

Because of the orthogonality of the system, spatial and functional compartmentalization of information within a shared environment becomes possible, which makes controlled and sequential selective access to individual data sets realizable. Furthermore, DNA encapsulation in nanovesicles and their incorporation into a stable and water-resistant fiber matrix strengthens the storage platform's durability and stability. This enables a second layer of protection from mechanical stress and oxidative degradation, essential for warm data storage. It also serves to reduce reliance on technologically advanced outside-sorting systems for data retrieval.

To achieve a fully PCR-independent selection matrix, the scalability and multiplexing capacity of this system are essential. Within this work, we provide a preliminary proof of concept using two distinct orthogonal triggers. Future efforts will focus on expanding the trigger library by exploring pH gradients, combining pH response with the temperature response of lipids, as well as tuning the chemical properties of the polymers employed to create programmable and smart nanofibers.

At this stage, although our system does not yet offer the far-greater multiplexing potential of PCR-based approaches, its scalability can be tailored to the intended application and end-use requirements.

Together, these results provide the first foundations towards a chemically addressable and accessible non-PCR-based DNA memory architecture. We open a path to storing more complex information, and to exploring and creating a wider library of orthogonal stimuli-responsive vesicles and nanomaterials.

## Materials & methods

### Neutral SUV synthesis, embedding in electrospun nanofibers and characterization

2 : 3 Ratio of neutral lipids DMPC : DPPC, (1,2-ditetradecanoyl-*sn*-glycero-3-phosphocholine and 1,2-dihexadecanoyl-*sn*-glycero-3-phosphocholine) both obtained from Avanti Polar Lipids, were dissolved in chloroform to reach a 5 mg mL^−1^ final concentration, together with 1.5% mol DiO (3,3′-dioctadecyloxacarbocyanine perchlorate). The dye was obtained from Sigma-Aldrich. A round bottom flask was used for the lipid thin layer formation, using a vacuum pump and a manual stirrer to evaporate chloroform in the process. Once the thin layer was synthesized, it was hydrated in 1 mL Milli-Q water, on a stirring plate at 150 rpm, 50 °C for 2 h. The solution was kept at 4 °C overnight. The solution was then sonicated to obtain heterogeneous multilamellar vesicles, and extruded using an Avanti Research Mini-Extruder through 100 nm polycarbonate membranes. Excess DiO was removed using 0.4 mL 100 kDa Amicon Filters; the supernatant (SUVs) was resuspended to obtain the same final volume/concentration. The synthesized SUVs were characterized by DLS (Malvern-Zetasizer).

After SUV characterization, 200 mg PEO (10% w/w, *M*_w_ = 400 kDa, from Merck) was dissolved in 1.8 g Milli-Q water. Then, 0.2 mL of SUVs (*ca.* 10% v/v) was added and gently mixed for 30 min. The mixture was electrospun using an E-fiber EF100 electrospinning SKE system, applying the voltage at 10 kV, with a collector placed at a distance of 12 cm from the syringe needle, at 350 µl h^−1^ flow rate. The fiber mesh was collected and the yield was calculated. A sample of the fiber mesh was also collected on a microscope slide and observed by fluorescence microscopy (ZEISS Axio Imager 2, light source Colibri7, FK92 filter). For PEO-SUV fiber characterization, environmental scanning electron microscopy (ESEM) was used (FEI Quanta 250). Standard deviation was calculated for fiber diameter measurements.

### DNA encapsulation in DMPC : DPPC and DNA release

DNA1 was encapsulated in DMPC : DPPC vesicles. During the hydration step of SUV synthesis, 20 µl DNA1 100 µM was mixed into a final 1 mL Milli-Q water solution. The rest of the synthesis steps were followed in the same way. After formation of homogeneous DNA-containing SUVs, the remaining non-encapsulated DNA was removed, using 0.4 mL 100 kDa Amicon filters. Encapsulation efficiency was calculated using the initial DNA quantity added in the hydration solution and the free DNA after Amicon filtering (Formula S1), measured by absorbance at 260 nm (NanoDrop 2000 spectrometer, Thermo Fisher Scientific).

Next, two fiber meshes were created. First, 200 mg PEO (10% w/w, *M*_w_ = 400 kDa) were dissolved in 1.8 g Milli-Q water, containing 4 µl of DNA1 100 µM (0.0015% w/w). Second, 200 mg PEO (10% w/w, *M*_w_ = 400 kDa) were dissolved in 1.8 g Milli-Q water, and 0.2 mL of SUVs (*ca.* 10% v/v) were added and gently mixed for 30 min. The electrospinning conditions were set at 10 kV, with the syringe needle to 12 cm distance from the collector, at 350 µl h^−1^ flow rate. The retrieval of the DNA from “naked”-DNA-fibers and DNA from SUV-DNA-fibers was compared.

Each fiber mesh sample was solubilized in water (70 mg sample per mL water). 5 µl from each sample were taken at time = 0 and after 24 h at room temperature. After 24 h, the samples were heated at 37 °C for 1 h, centrifuged at 20 000 g for 20 min and 5 µl from supernatant were kept for PCR. Next, both samples were heated to 80 °C for 1 h. Finally, 0.5% Triton X-100 (from Sigma-Aldrich) was added for 30 min to PEO-SUV sample, and the released DNA was purified using desalting columns. Lastly, PCR amplification of each sample was performed and analyzed in agarose gel electrophoresis.

### Desing of pH responsive vesicles and release studies

For acidic pH-responsive SUVs, a ratio of 3 : 2 of DOPE : CHEMS (1,2-di-(9*Z*-octadecenoyl)-*sn*-glycero-3-phosphoethanolamine and 3β-hydroxy-5-cholestene 3-hemisuccinate, from Avanti Polar Lipids) was prepared, and for alkaline pH-responsive SUVs, a ratio of 3 : 2 DSPA (1,2-dioctadecanoyl-*sn*-glycero-3-phosphate, from Avanti Polar Lipids) : DPPC lipids was prepared. Both SUV sets were prepared following the same procedure used for neutral vesicles. These vesicles were characterized in solution using DLS, immersing each sample in pH 5.5 acetate buffer, pH 7.4 PBS and pH 10 (0.1 mM) NaOH solutions.

After characterization, DNA1-loaded DOPE : CHEMS vesicles and DNA2-loaded DSPA : DPPC vesicles were synthesized following the same protocol. In each case, 20 µl DNA 100 µM was mixed into a final 1 mL Milli-Q water, in the hydration step. The encapsulation efficiencies were calculated, and both loaded SUVs were characterized by DLS under each pH condition. For DLS analysis, standard deviations of all samples were calculated. Samples from each DNA-loaded SUV solution from each pH were taken to test DNA release. For this, PCR amplification (specific for each DNA) and agarose gel electrophoresis were run.

To test selective retrieval in solution, a 1 mL solution was prepared using a 50 : 50 mixture of each stock loaded-vesicle solution. The DNA release was tested after obtaining a 1/10 dilution of the mix in pH 5.5 acetate buffer and 1/10 dilution of the mix in pH 10 NaOH solution, keeping each pH constant.

From each step, two aliquots (each in triplicate) were taken, and each one was subjected to each of the primer sets for the PCR verification step of the released DNA, and analyzed by agarose gel electrophoresis.

### Testing SUV and polymer stability

The SUV solutions were incubated under a 254 nm UV lamp at time points of 30 min, 1 h, 2 h, 3 h and 4 h. SUV stability in solution was checked over time by DLS.

In a separate experiment, a polymer solution composed of 160 mg of PEO (*ca.* 8% w/w, 400 kDa), 68.6 mg of TMPTA (70 : 30 = PEO : TMPTA, *M*_w_ = 296 Da) and 2 mg of benzophenone (1.2 wt% *vs.* PEO) was prepared in a final 1.8 g of Milli-Q water, and electrospun at 12 kV, with the syringe needle at 12 cm from the collector, at 300 µl h^−1^ flow rate. The optimum and effective cross-linking time under UV exposure was also determined after observing the water resistance of the fiber mesh. UV cross-linking was performed using a Spectroline E-Series 4 W UV lamp (Spectronics Corporation, USA; 230 V, 50 Hz, 0.17 A) at 254 nm. The meshes were irradiated under ambient conditions. TMPTA and benzophenone were also purchased from Merck.

### Encapsulation of pH responsive SUVs in electrospun nanofibers

On one hand, a polymer solution composed of 160 mg of PEO (*ca.* 8% w/w, 400 kDa), 68.6 mg of TMPTA (70 : 30 = PEO : TMPTA) and 2 mg of benzophenone (1.2 wt% *vs.* PEO) was prepared in a final 1.8 g of Milli-Q water. Once solubilized, 0.2 mL (*ca.* 10% v/v) DNA1-loaded DOPE : CHEMS (after filtration) was added, and incubated for 45 min under slow stirring, for a homogeneous mixture. The final solution was electrospun under 12 kV, 0.2 mL h^−1^, with the syringe needle at 12 cm distance from the collection plate, and the fiber mesh was exposed to UV for 2 h, post-electrospinning.

First, the fiber mesh (70 mg sample per mL water) was immersed in pH 7.4 1× PBS solution, a sample was taken, and the mesh was dried. Next, the mesh was immersed in the solution where no release was expected, based on DNA release in solution experiments. In this case, it was pH 10 NaOH; a sample was taken, and the mesh dried. Finally, the fiber cut was immersed in pH 5.5 acetate buffer and a sample was taken. The samples were taken in triplicate.

On the other hand, the same polymer solution was prepared, this time only adding 0.2 mL (10% v/v) DNA2-loaded DSPA : DPPC, following the same electrospinning procedure. This time, the fiber mesh was first immersed in pH 7.4 1× PBS followed by pH 5.5 acetate buffer and pH 10 NaOH immersions. The samples were taken in triplicate.

Finally, all the samples were PCR-amplified, where the samples from the first mesh were subjected to PS1 (primer set 1), while the samples from the second fiber mesh were subjected to PS2 (primer set 2). DNA presence/absence was checked by agarose gel electrophoresis.

### Random access: experimental protocol

10% v/v (*ca.*) DNA1-DOPE : CHEMS and 10% v/v (*ca.*) DNA2-DSPA : DPPC were mixed in the same cPEO polymer solution, after re-calculating the quantities for a final 2 mL solution. The optimal electrospinning conditions were 0.3 mL h^−1^ flow rate, 12 kV potential, and 12 cm distance from the syringe needle to the collector plate. Conditions for the cross-linking were kept the same.

To test the orthogonal random access and fiber sequential retrieval, a full experiment was proposed, and its results were verified in two steps: PCR amplification of the released DNA and agarose gel electrophoresis (PCR in this case is only for verification, not for random access), and sequencing and decoding of the retrieved DNA from the previous step.

First, the fiber mesh (70 mg mL^−1^ cut) was immersed in pH 7.4 1× PBS and two aliquots were taken. To one aliquot PS1 was added, and to the other one PS2. Next, the fiber mesh was dried, washed and immersed in pH 10 NaOH. Again, two aliquots were taken. To one, PS1 was added and to the other one PS2. Finally, the fiber mesh was again dried, washed and immersed in pH 5.5 acetate buffer, and again two aliquots were taken. To one, PS1 was added and to the other one PS2. All the samples were taken in triplicate.

To check the orthogonality, the exact same experiment was performed but reversing the order of immersion in different pH solutions: pH 7.4 → 5.5 → 10. All the aliquots were subjected to PCR amplification, and the presence/absence of released DNA was tested in agarose gel electrophoresis. As a final step, the samples were sent for sequencing.

### PCR amplification and agarose gel electrophoresis

The PCR-amplification verification step was carried out using a MiniAmpTM plus thermocycler (Applied Biosystems, Thermo Fisher Scientific). The reactions were performed using 0.1–5 ng DNA template or 5 µl release-samples, using 0.5 µM final concentration of each of the forward and reverse primers, and 200 µM final concentration of dNTP mix, using OneTaq HotStart, HiFi polymerase (New England Biolabs), in a final volume of 50 µl. The PCR reaction cycle ran as follows: 94 °C for 30 s for initial denaturation, and next, 30 cycles for 1) denaturation at 94 °C for 10 s, 2) annealing at 65 °C for 30 s, 3) extension at 68 °C for 30 s, and finally, 3 min at 68 °C for final extension.

The final products were verified using 2% agarose (analytical grade Agarose LE, Thermofisher) gel electrophoresis, using 0.5× TBE pre-stained with SYBRSafe (10 000×, from Thermofisher). A 10 µl aliquot of each amplified sample was mixed with 2 µl of 6× DNA Gel Loading Dye (from Thermofisher) and loaded into each well. The electrophoresis was performed under 80 V. For reference, FastLoad 50 bp DNA Ladder (from SERVA) was used. Finally, the gels were checked with a Bio-Rad UV transilluminator.

### qPCR

qPCR reactions were carried out using a CFX Opus 96 Real-Time PCR System (Bio-Rad). The reactions were performed on 1 µL of DNA samples using 10 µL of Luna® Universal qPCR Master Mix (New England Biolabs), with forward and reverse primers at 250 nM final concentration in a reaction volume of 20 µL. A standard thermal protocol was performed according to the manufacturer's instructions (with annealing temperature: 63 °C). Relative sample quantification was performed by interpolation from a standard curve generated from standard DNA samples of known concentration. CFX Maestro software version 2.3 (Bio-Rad) was used to perform baseline correction and calculate the quantification cycles (*C*_q_) and relative concentrations.

### DNA sequencing

Sanger sequencing was performed on released DNA samples in each condition, as well as on the reference DNA samples. These samples were purified using a dNEAT PCR Purification Kit (from Labbox) and sequenced (sent to Stab Vida, Caparica, Portugal). The results were processed and analyzed using CodonCode Aligner software.

### 24-Hour high-temperature stress test

A humidity chamber was prepared using an Eppendorf-tube heater set at 85 °C next to a plate with saturated sodium chloride solution. This setup was placed inside a box and the relative humidity was measured, until it established a constant value of around 50%. The fiber samples were incubated for 24 h inside this set-up, and next, random access experiments were performed (see “random access: experimental protocol” section).

## Author contributions

The manuscript was written through contributions of all authors. All authors have given approval to the final version of the manuscript.

## Conflicts of interest

There are no conflicts to declare.

## Supplementary Material

NA-008-D6NA00595K-s001

## Data Availability

The data supporting this article have been included as part of the supplementary information (SI). Supplementary information: DLS data, fluorescence microscopy images, additional gel electrophoresis images, qPCR data, DNA sequence designs, and formulas. See DOI: https://doi.org/10.1039/d6na00595k. The Sanger sequencing data generated, including raw chromatogram files, are available at: https://doi.org/10.5281/zenodo.22903328.
