# Supplementary material for: Stimuli-responsive vesicles in water-resistant nanofibers for molecular random access in DNA data storage
Source: Nanoscale Adv. 2026 Sep 21;8(21):6382–92. doi: 10.1039/d6na00595k (PMC13632630; doi:10.1039/d6na00595k)
Supplement: Supplementary file 1 [file NA-008-D6NA00595K-s001.pdf]

Supplementary Information (SI) for Nanoscale Advances.
This journal is © The Royal Society of Chemistry

Supporting Information

Stimuli-Responsive Vesicles in Water-Resistant Nanofibers for Molecular Random Access in DNA Data Storage

Jokin Yeregui-Elosua [a,b], Diana Soukarie [a‡], Cecilia Wetzl [a‡], Alexander M. Bittner [a,c], Ibon Santiago [a]*

[a] CIC nanoGUNE BRTA, Donostia-San Sebastián, 20018, Spain

[b] University of the Basque Country, EHU, Donostia-San Sebastián, 20018, Spain

[c] IKERBASQUE, Basque Foundation for Science, 48009, Bilbao, Spain

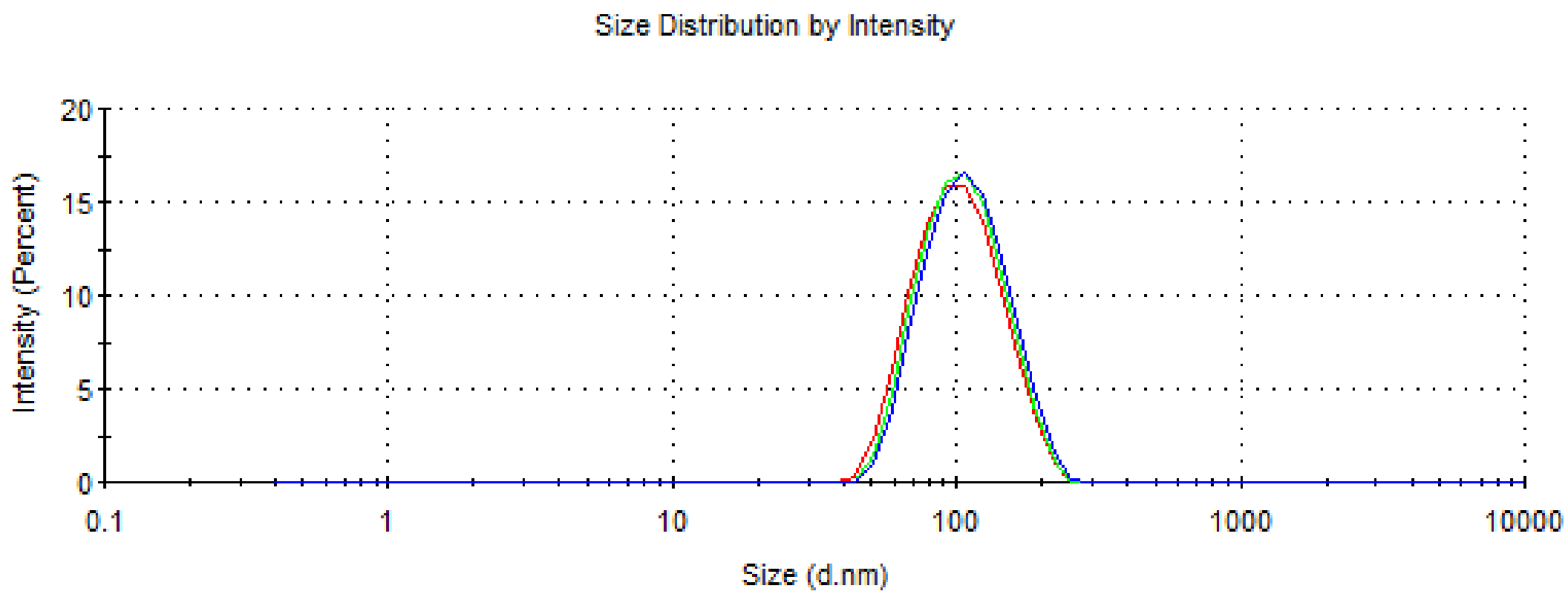

Figure S1. Size distribution by Intensity of synthesized SUVs measured with DLS Zetasizer. The intensity peak is observed at 100 nm diameter size, suggesting monodispersity and a homogeneously sized sample, also supported by the polydispersity index (PDI).

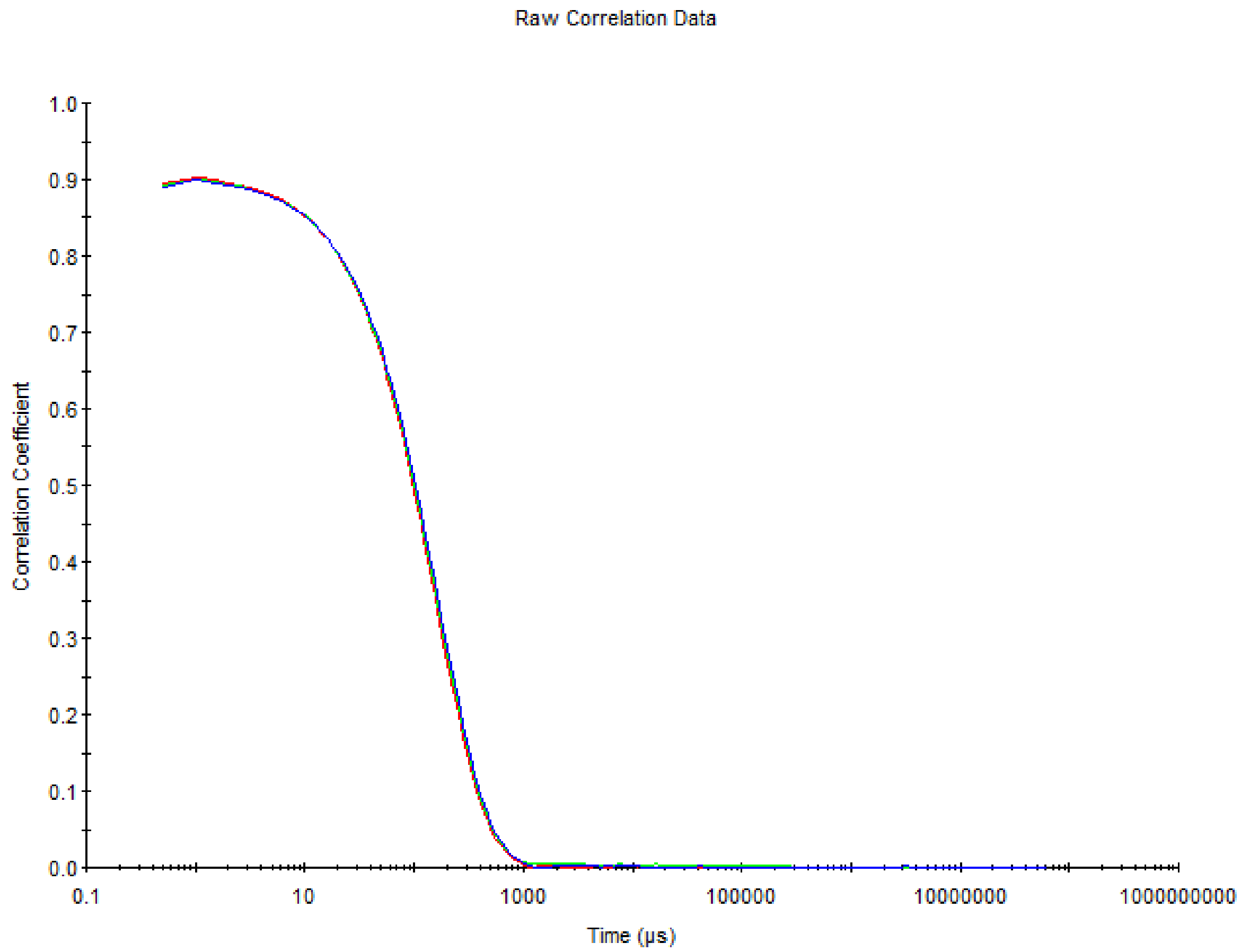

Figure S2. Raw autocorrelation data from DLS showing the intensity autocorrelation function versus delay time. This single exponential decay represents a very monodisperse sample with low noise and good signal quality.

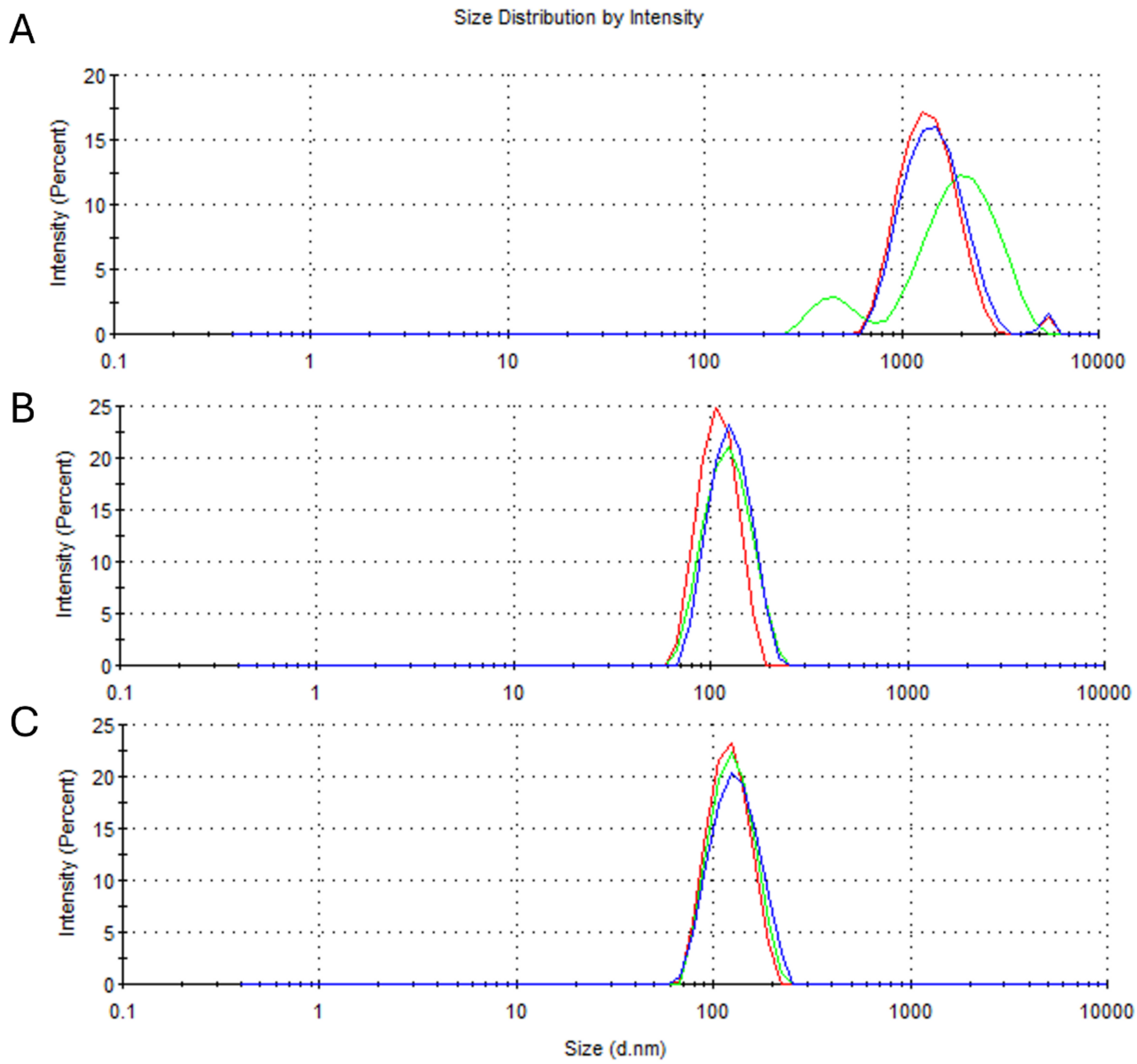

Figure S3. Size distribution by Intensity of synthesized DOPE:CHEMS SUVs, measured with DLS Zetasizer. A) Vesicle diameter distribution when immersing the samples in pH 5.5 acetate buffer. **B)** Vesicle diameter distribution when immersing the samples in 1X pH 7.4 PBS solution. **C)** Vesicle diameter distribution when immersing the samples in pH 10 NaOH solution.

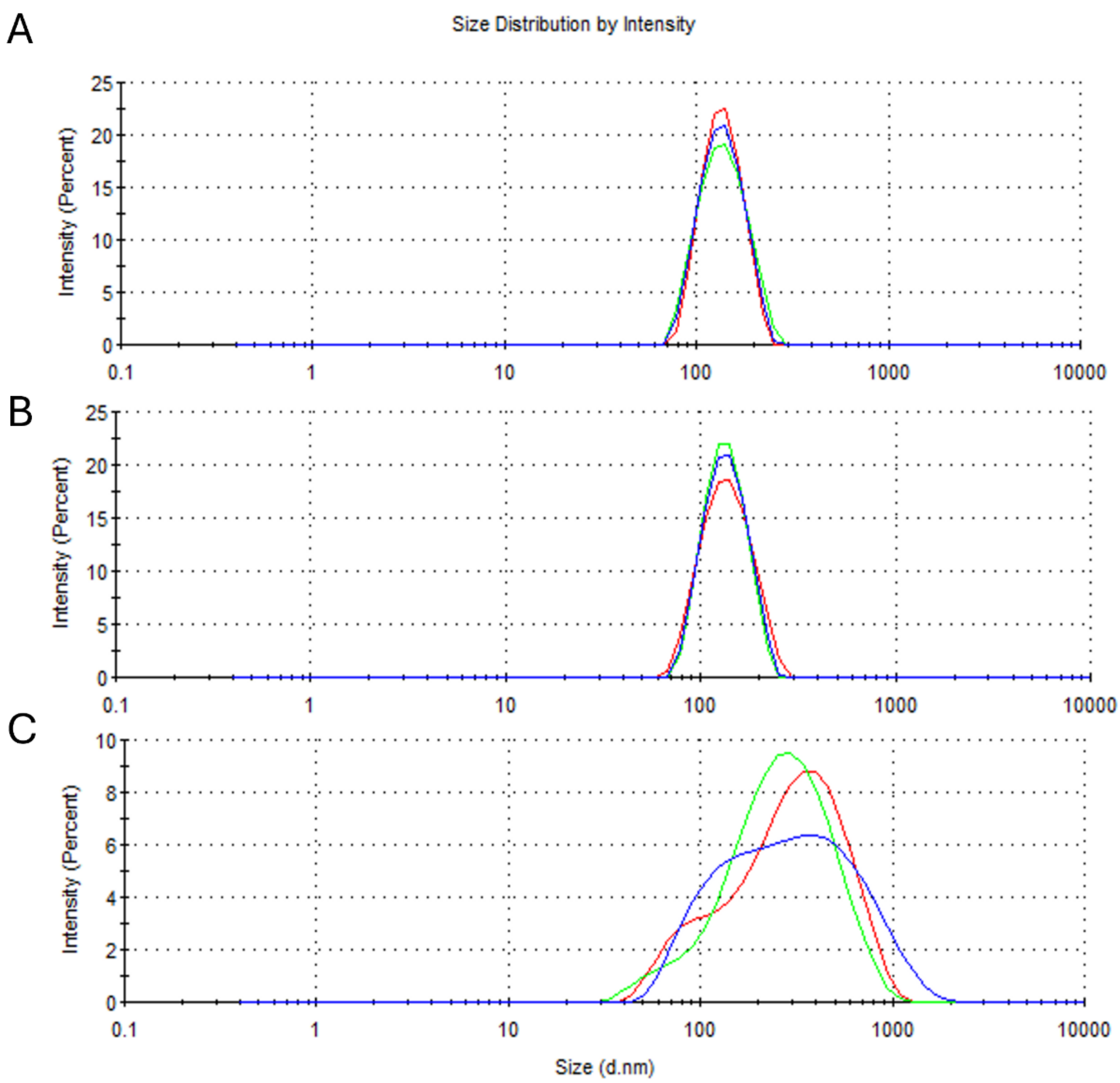

Figure S4. Size distribution by Intensity of synthesized DSPA:DPPC SUVs measured with DLS Zetasizer. **A)** Vesicle diameter distribution when immersing the samples in pH 5.5 acetate buffer. **B)** Vesicle diameter distribution when immersing the samples in 1X pH 7.4 PBS solution. **C)** Vesicle diameter distribution when immersing the samples in pH 10 NaOH solution.

DNA1 data: “Nanogune” → “Nanogune Nanogune” reiteration

5’TCCTCTACAGAGAGCAGGTT**CGCC**ACTGAGCAAGTGAGTTAGATATAAAGTGAGAA**CGCC**
ACTGAGCAAGTGAGTTAGATATAAAGTGAGAATACCTGTCTCGAAGTTGCGT3’

DNA2 data: “Textadna” → “Textadna Textadna” reiteration

5’TCGTAGCACTTGACGATACT**CGCC**AAACAGAAATGCATACAGCAAGACAGTGAGCA**C**
GCCAAACAGAAATGCATACAGCAAGACAGTGAGCAACGCAACTTCGAGACAGGTA3’

Figure S5. DNA strand design for data-encoding DNA synthesis. In gray, the forward and reverse primer binding sites; in green, the payload of the encoded data (reiteration); in bold, “CGCC” spacers, separating each reiteration. The ssDNAs are 112 nt long.

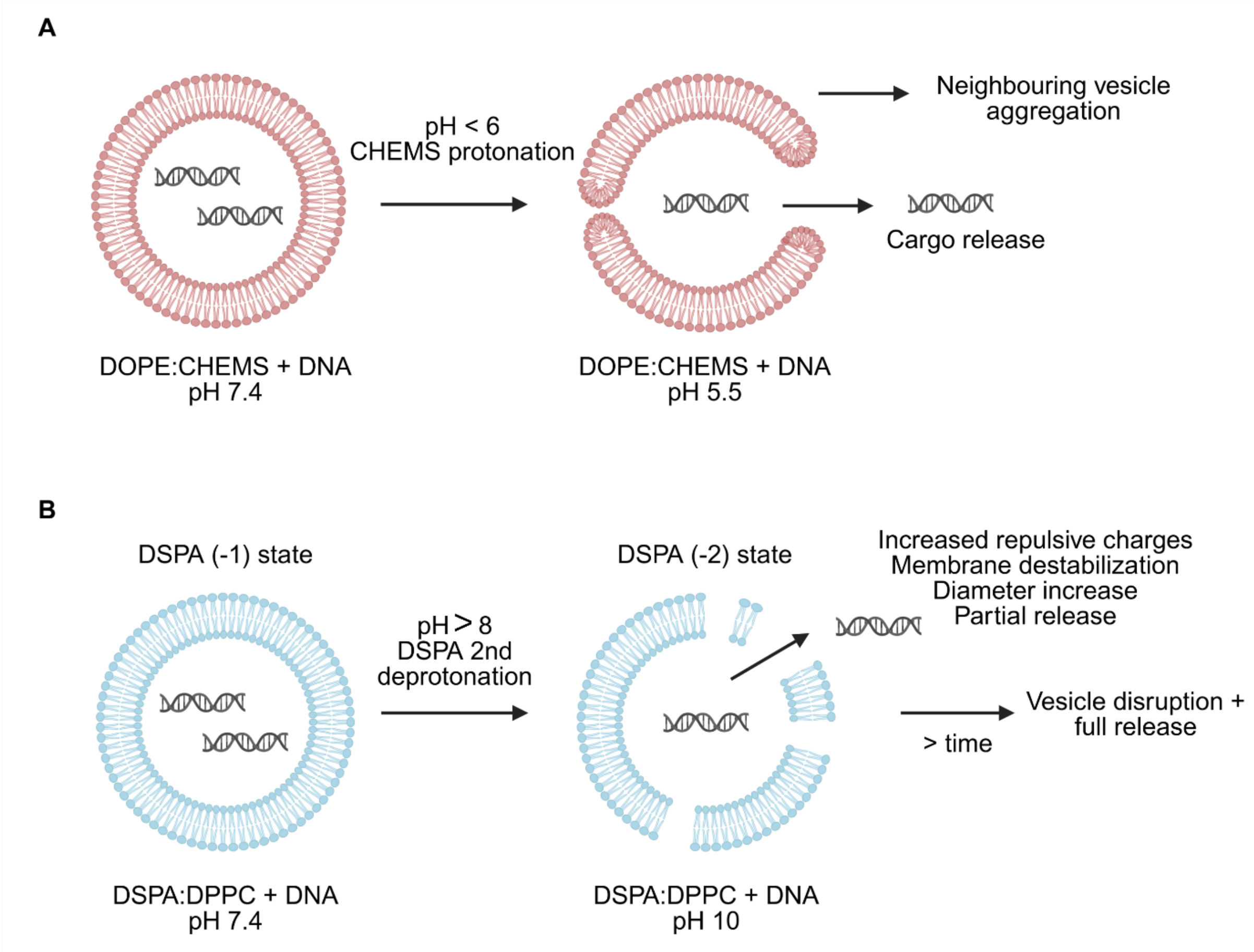

Figure S6. Schematic representation of orthogonal pH-responses of triggered vesicles. A) Schematic representation of DOPE:CHEMS vesicles' response to acidic pH. A decrease in pH would trigger the structural conformation shift of the compound, causing target release and an aggregation-prone state. **B)** Hypothetical schematic representation of alkalinity-responsiveness of DSPA:DPPC vesicles. An increase in pH would trigger the overall charge state imbalance and membrane destabilization, causing possible target release and disruption of the vesicles.

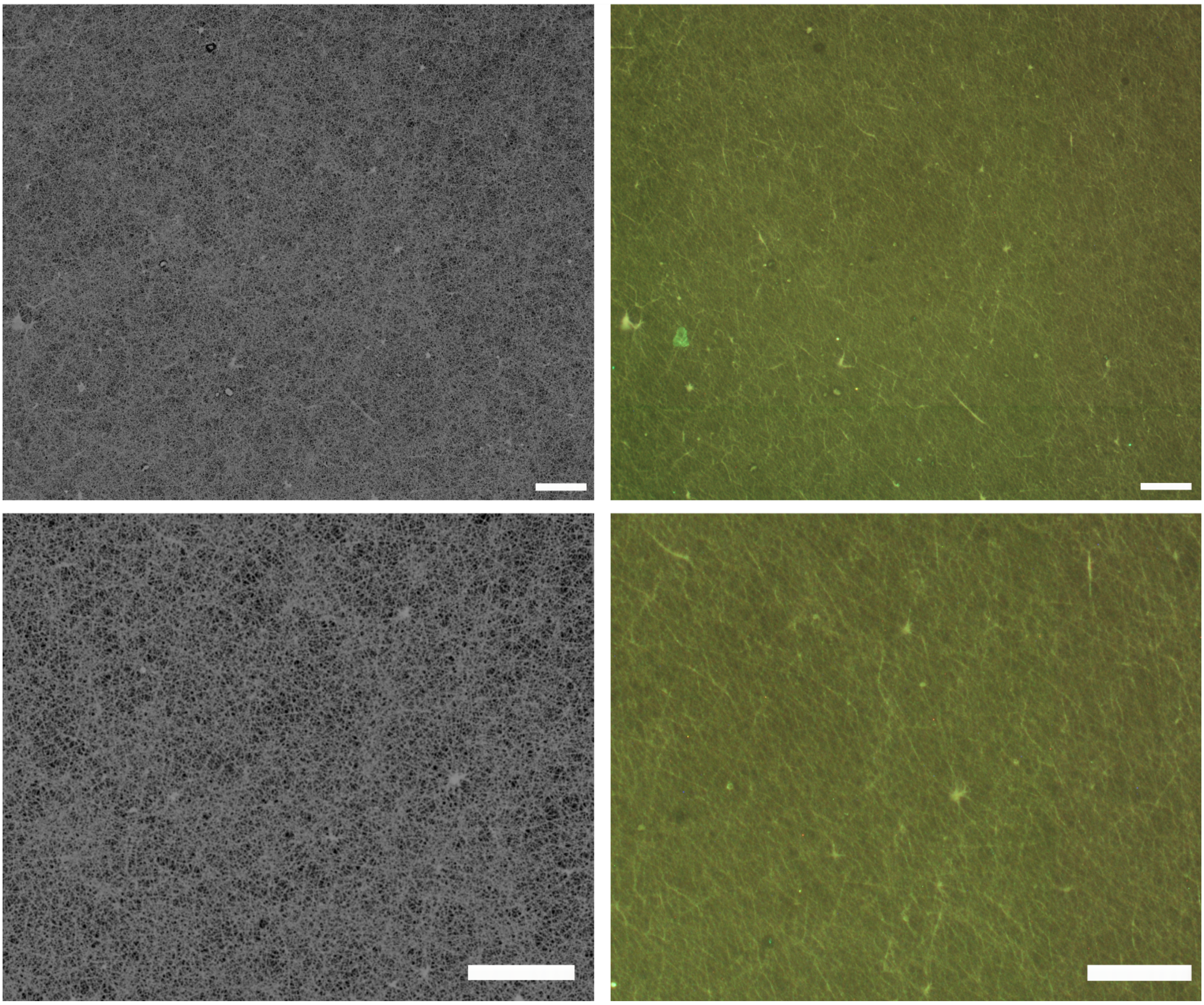

Figure S7. Fluorescence microscopy images of fluorescently labeled SUVs embedded in a thin electrospun nanofiber mesh at lower magnification. Bright-field images: zoomed-out image (top left) and zoomed-in image (bottom left), same region. Fluorescence images: zoomed-out image (top right) and zoomed-in image (bottom right), same region. The scale bar is 100 μm.

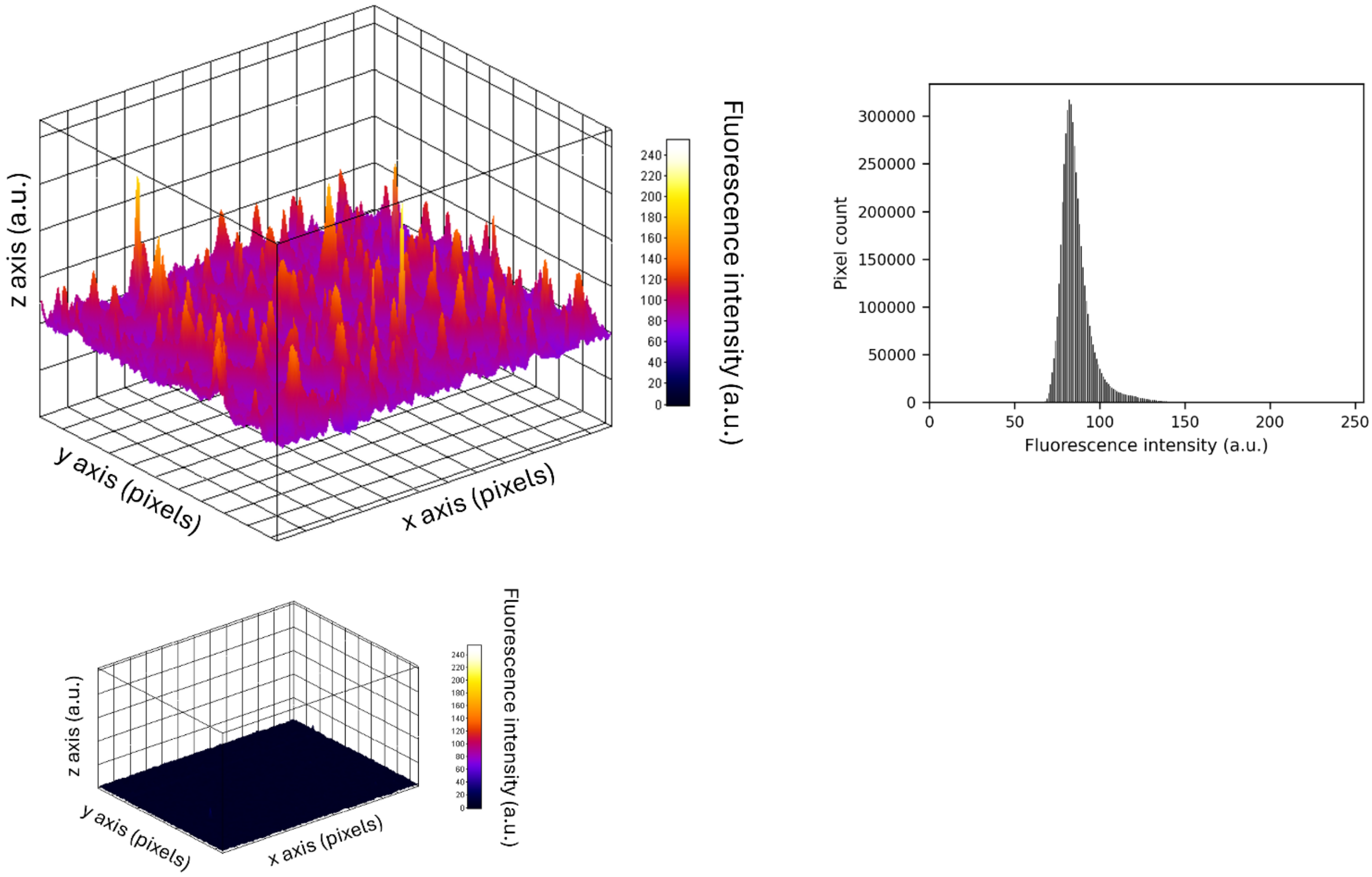

Figure S8. 3D profiling map and histogram of the fluorescence intensity distribution of the thin fiber mesh sample in Figure 3. On the top left, the 3D fluorescence intensity surface profile. On the right, the corresponding pixel-wise intensity histogram. Bottom left figure is a negative control. The measurements were made after outlier removal. The surface plot highlights localized intensity regions, while the histogram gathers the global distribution of fluorescence values and the dominant signal range within the image.

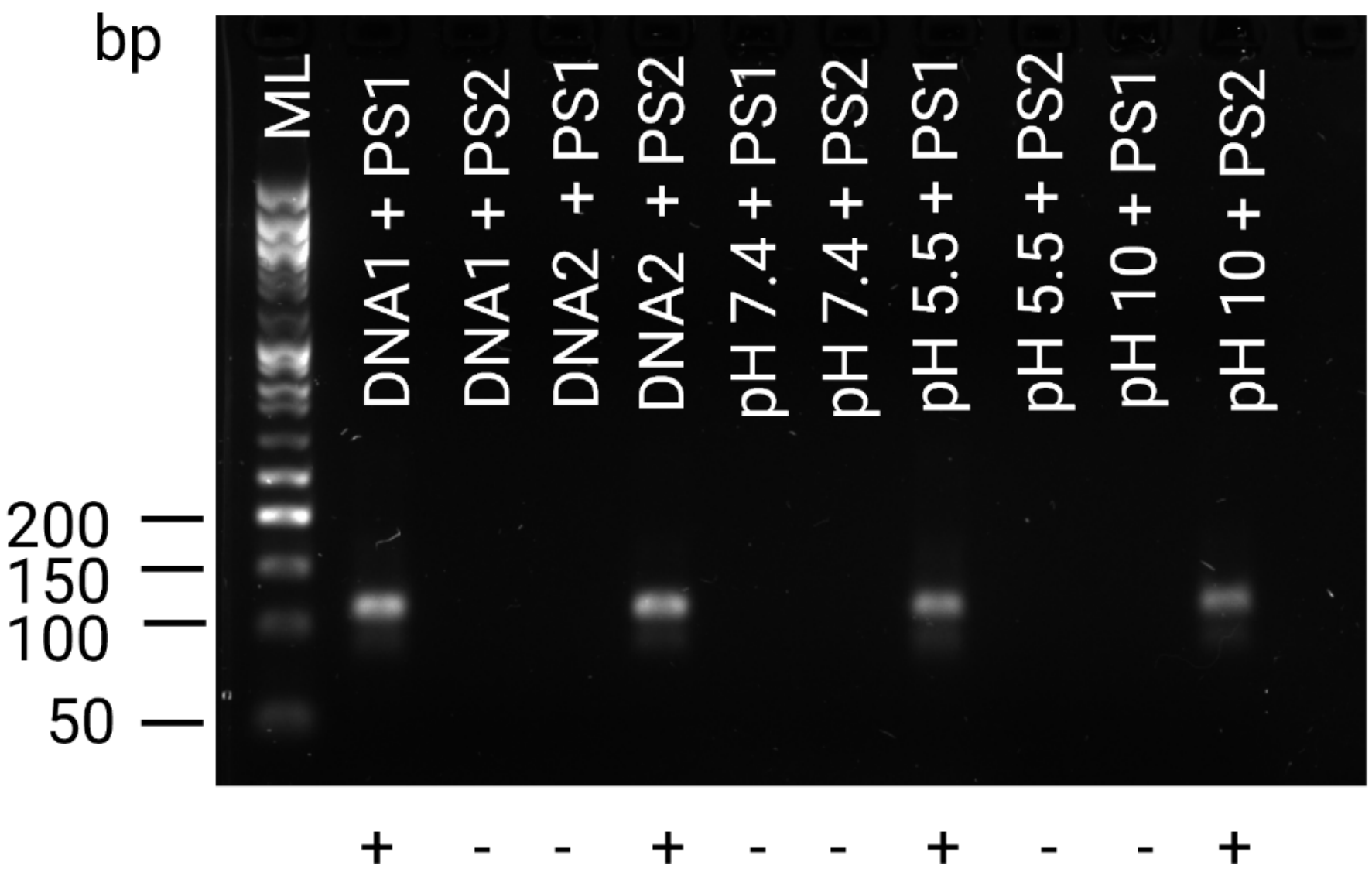

Figure S9. Agarose gel electrophoresis results of the proposed random access in solution experiment. 2% agarose gel electrophoresis after verification-PCR. From left to right: DNA marker ladder (50 bp); DNA1 positive control with PS1; DNA1 negative control with PS2; DNA2 negative control with PS1; DNA2 positive control with PS2; sample from pH 7.4 immersion with PS1; sample from pH 7.4 immersion with PS2; sample from pH 5.5 immersion with PS1; sample from pH 5.5 immersion with PS2; sample from pH 10 immersion with PS1; sample from pH 10 immersion with PS2. The lower bar: + DNA presence (after PCR); - DNA absence (after PCR).

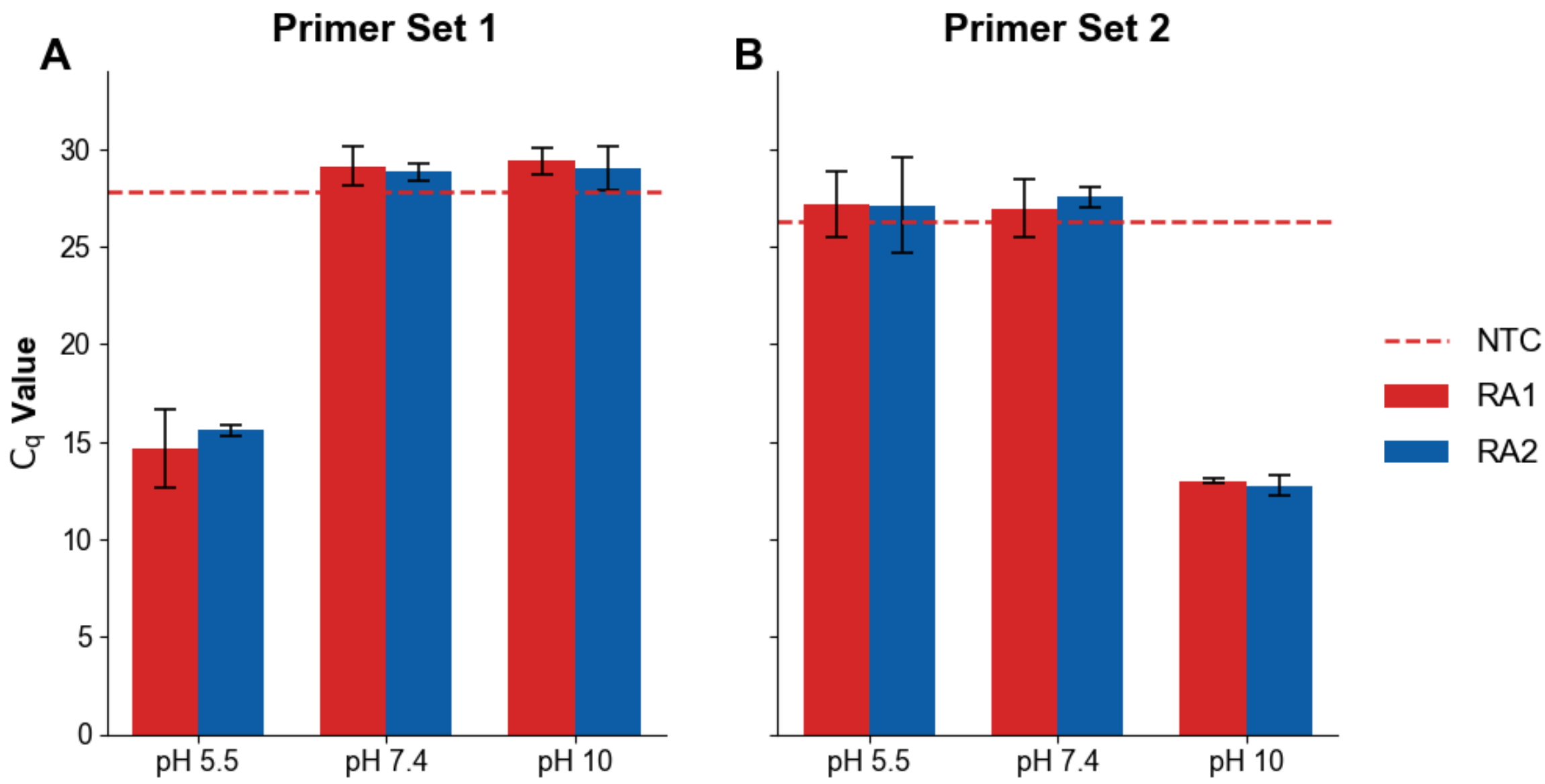

Figure S10. **Real-time qPCR amplification Cq values under varying pH triggers.** Evaluation of quantification cycle (Cq) values across two orthogonal setups using **(A)** Primer Set 1 (PS1) targeted toward DNA1 and **(B)** Primer Set 2 (PS2) targeted toward DNA2. RA1 refers to the first sequential experiment (pH 7.4 à pH 5.5 à pH 10 immersion cycle) and RA2 to the second one (pH 7.4 à pH 10 à pH 5.5 immersion cycle). The Cq values and released DNA amounts have an inverse logarithmic relationship: low Cq, high initial DNA concentration; high Cq, low initial DNA concentration. Red dashed lines indicate the no-template control (NTC) baseline for each respective primer configuration. **(A)** Subjecting each sample to PS1, high Cq values ($\sim$ 28) matching the NTC

are observed at pH 7.4 and pH 10, indicating no detectable amplification, while a sharp drop in Cq ($\sim$ 15) at pH 5.5 confirms robust, selective DNA1 amplification. **(B)** Subjecting each sample to PS2, high Cq values ($\sim$ 26) matching the NTC are observed at pH 5.5 and pH 7.4, indicating no detectable amplification, while a sharp drop in Cq ($\sim$ 12) at pH 10 confirms robust, selective DNA2 amplification. Data represents mean ± standard deviation (n = 3 independent replicates).

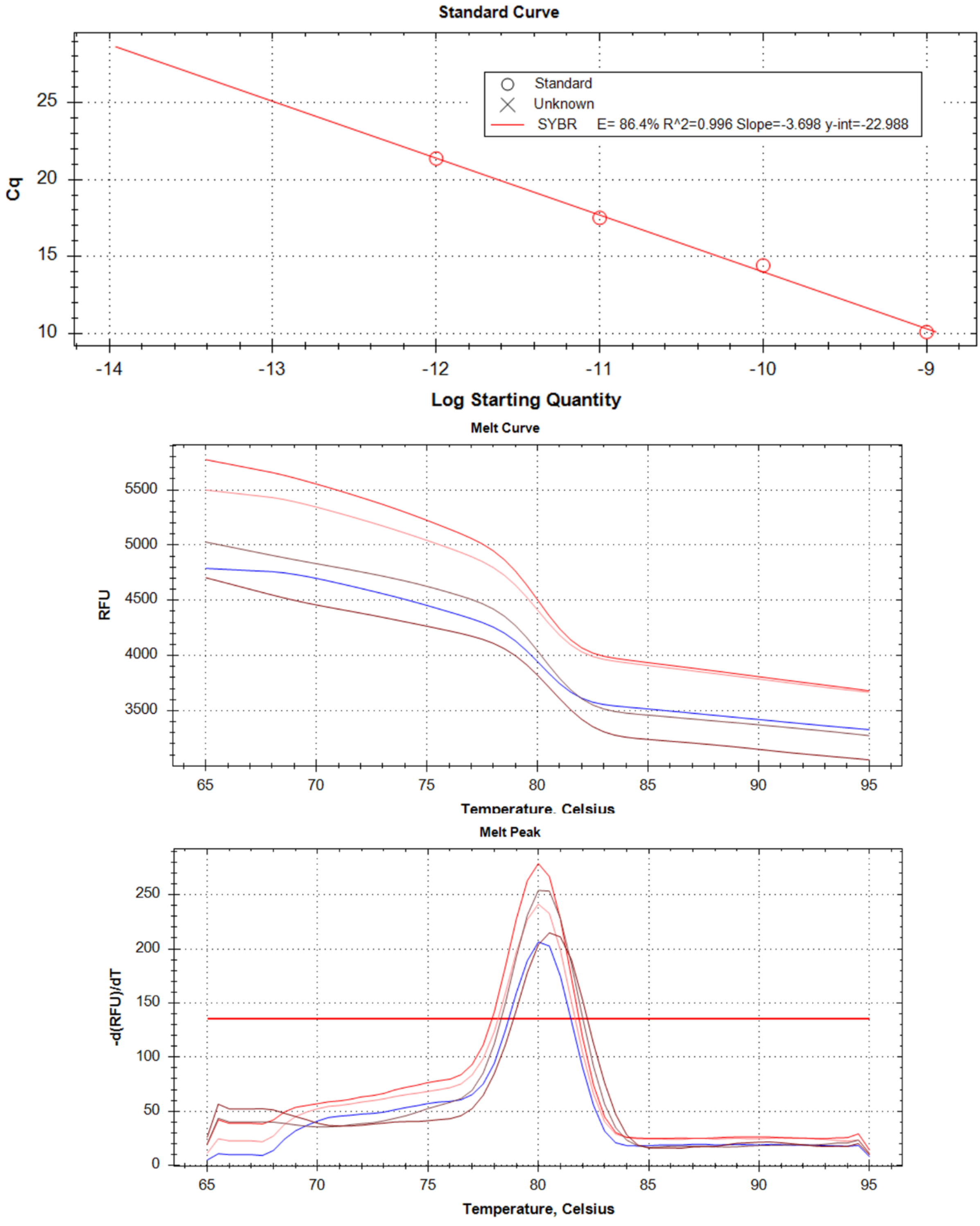
Standard Curve
Standard
Unknown
SYBR E= 86.4% R^2=0.996 Slope=-3.698 y-int=-22.988
Cq
25
20
15
10
-14
-13
-12
-11
-10
-9
Log Starting Quantity
Melt Curve
RFU
5500
5000
4500
4000
3500
65
70
75
80
85
90
95
Temperature, Celsius
Melt Peak
-d(RFU)/dT
250
200
150
100
50
0
65
70
75
80
85
90
95
Temperature, Celsius

A

B

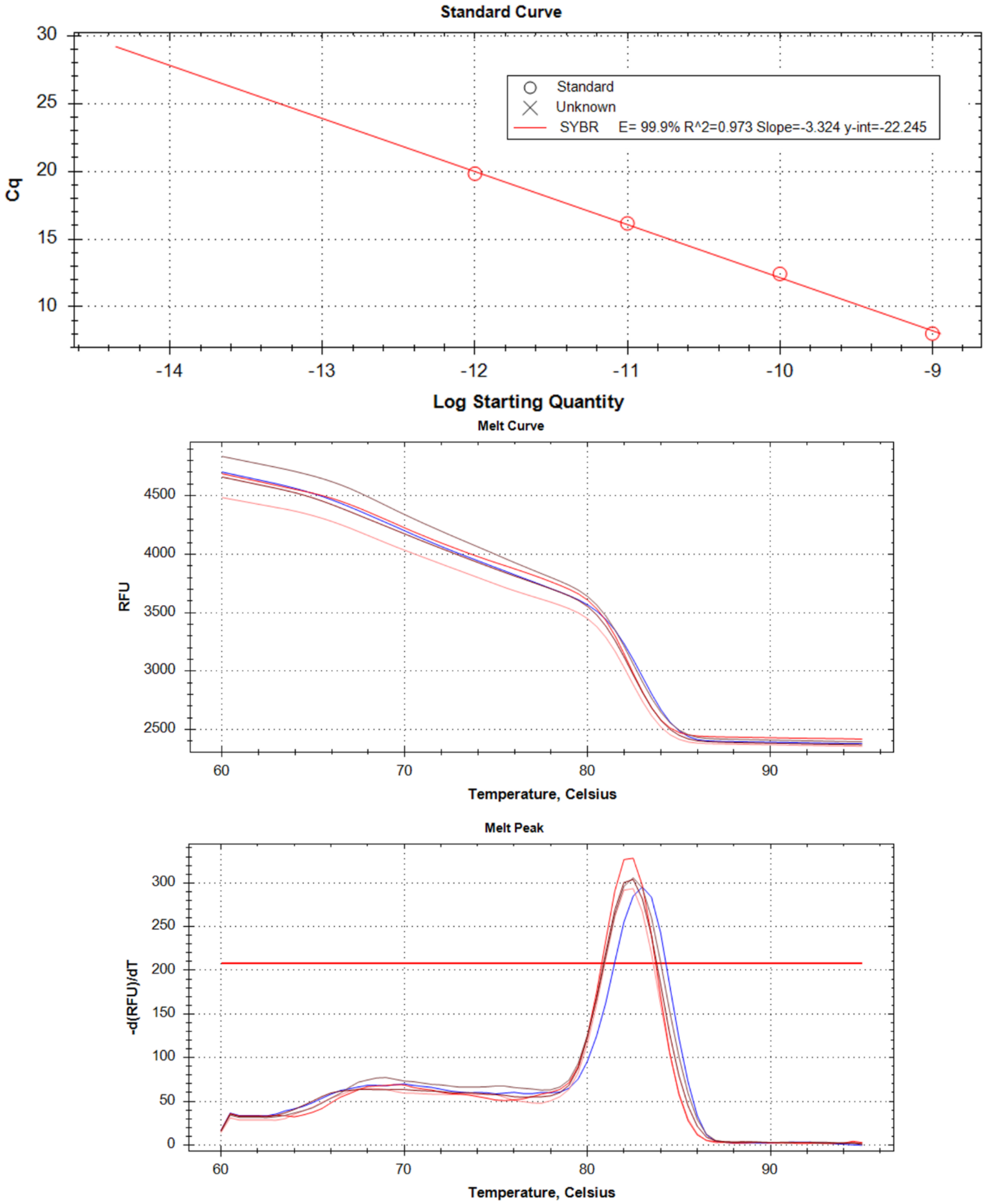

Standard Curve
Cq
Log Starting Quantity
Standard
Unknown
SYBR E= 99.9% R^2=0.973 Slope=-3.324 y-int=-22.245
30
25
20
15
10
-14
-13
-12
-11
-10
-9
Melt Curve
RFU
4500
4000
3500
3000
2500
60
70
80
90
Temperature, Celsius
Melt Peak
-d(RFU)/dT
300
250
200
150
100
50
0
60
70
80
90
Temperature, Celsius

C

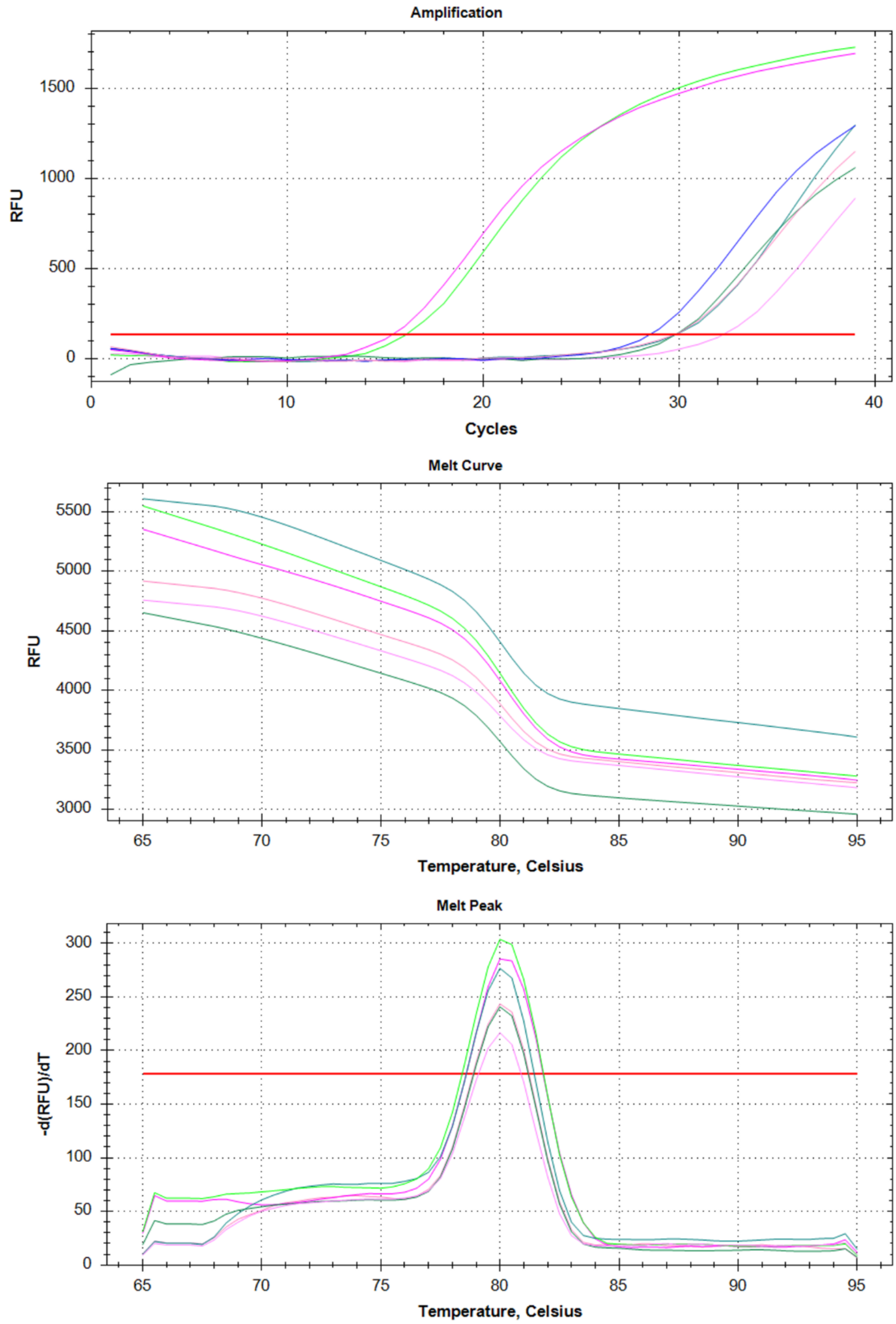

Amplification
RFU
1500
1000
500
0
0
10
20
30
40
Cycles
Melt Curve
RFU
5500
5000
4500
4000
3500
3000
65
70
75
80
85
90
95
Temperature, Celsius
Melt Peak
-d(RFU)/dT
300
250
200
150
100
50
0
65
70
75
80
85
90
95
Temperature, Celsius

D

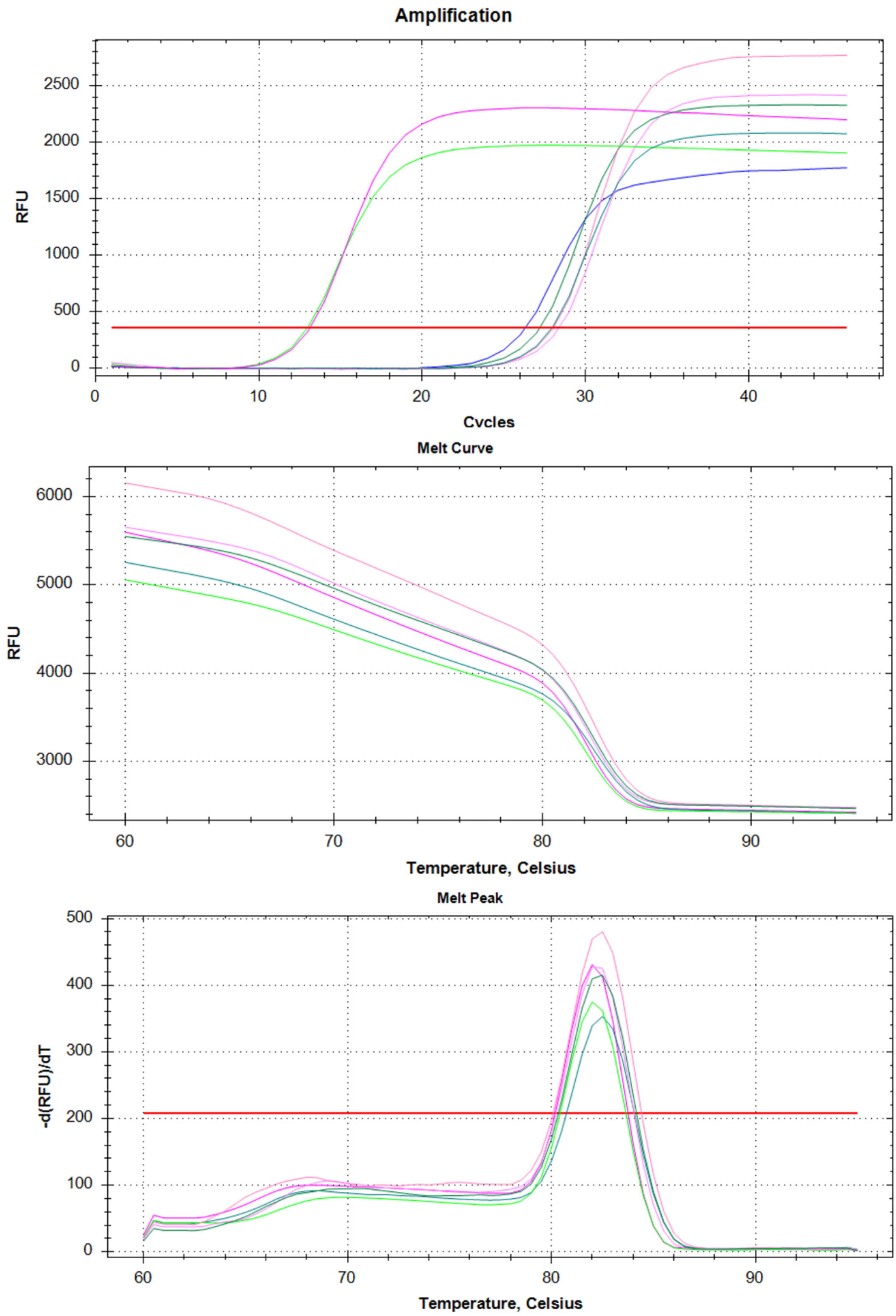

Amplification
RFU
2500
2000
1500
1000
500
0
0
10
20
30
40
Cycles
Melt Curve
RFU
6000
5000
4000
3000
60
70
80
90
Temperature, Celsius
Melt Peak
-d(RFU)/dT
500
400
300
200
100
0
60
70
80
90
Temperature, Celsius

Figure S11. qPCR standard curves and amplification profiles for the sequential DNA-release experiments. (**A**) Standard curve for primer set 1, including the no-template control (NTC) in the melting curves/ peak. Red shades denote the DNA standards, from darkest to lightest: 1 nM, 100 pM, 10 pM, and 1 pM. (**B**) Standard curve for primer set 2, including the NTC in the melting curves/ peaks; the red color palette denotes the same concentrations in the same order. (**C**) Amplification profiles of samples analyzed with primer set 1. Green shades represent samples from RA1, pink shades represent samples from RA2, and blue represents the NTC. RA1 refers to the first sequential experiment (pH 7.4 □ pH 5.5 □ pH 10 immersion cycle) and RA2 to the second one (pH 7.4 □ pH 10 □ pH 5.5 immersion cycle). Within each RA color palette, the most intense trace corresponds to the sample from the pH 5.5 immersion step, which showed specific DNA1 amplification and was used for comparative quantification; the remaining traces correspond to non-triggered conditions and were indistinguishable from the NTC. (**D**) Amplification profiles of samples analyzed with primer set 2, using the same sample color scheme. Within each RA color palette, the most intense trace corresponds to the sample from the pH 10 immersion step, which showed specific DNA2 amplification; similarly, the remaining traces correspond to non-triggered conditions and were indistinguishable from the NTC. The qPCR values are used to compare pH-specific release.

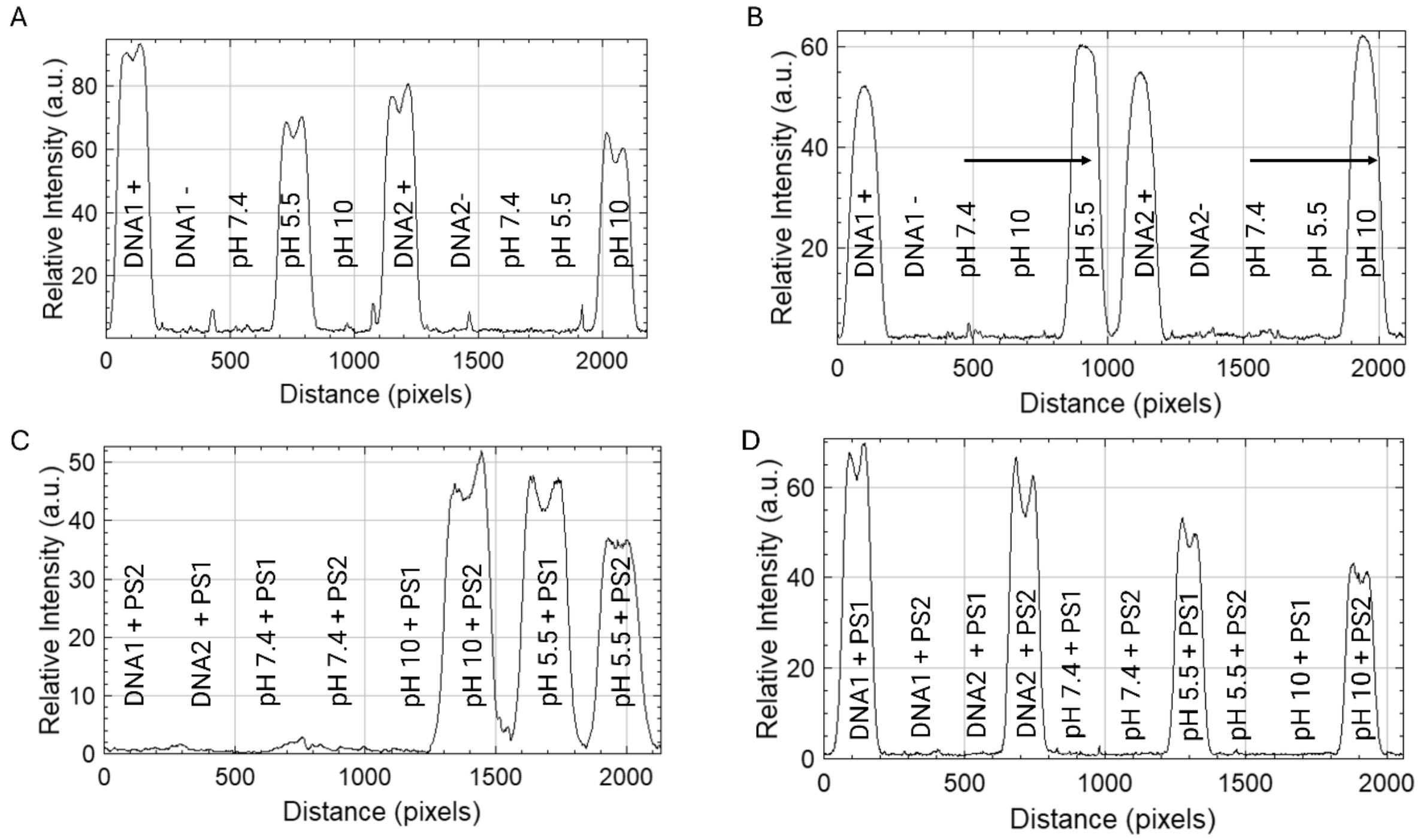

Figure S12. Densitometry data on plot profiling of agarose gel bands. From A to D, the plot profiling of gels in Figure 2, Figure 6, Figure 9, and Figure S9, respectively. The high intensity peaks reveal amplifiable released DNA in the medium, while the low intensity values refer to insufficient amplifiable DNA in the sample.

Table S1. DNA1 and DNA2 primer design. Each primer binds the primer-binding sites shown in Figure S5. Primer set 1 for the DNA1 binding sites, and primer set 2 for the DNA2 binding sites. These are used for the verification-step PCR, only to check which DNA is released using molecular orthogonal random access, in each specific condition. This PCR is not used for random access or selection.

(5’ □ 3’)	**Primer set 1**	**Primer set 2**
**Forward primer**	TCCTCTACAGAGAGCAGGTT	TCGTAGCACTTGACGATACT
**Reverse primer**	ACGCAACTTCGAGACAGGTA	TACCTGTCTCGAAGTTGCGT

Table S2. Empty DSPA:DPPC vesicle size distribution (in solution) after different exposure times under UV 254 nm. Stability was observed up to 2 h of exposure, where diameter size was relatively similar to t=0 parameters. Values of PDI < 0.1 are desired for highly monodisperse samples, while PDI > 0.2 indicates polydisperse samples.

Time (h)	Av. Diameter (nm)	PDI
0.5	139.9 ± 3.9	0.07 ± 0.02
1	140.7 ± 4.8	0.08 ± 0.03
2	155.3 ± 3.5	0.10 ± 0.01
**3**	**220.6 ± 85.1**	**0.23 ± 0.03**

$$\text{Enc. Efficiency (\%)} = \frac{\text{Initial DNA } (\mu g) \text{ - Non enc. free DNA } (\mu g)}{\text{Initial DNA } (\mu g)} \times 100$$

where

$$\text{Non enc. free DNA } (\mu g) = [C]_{drop} \ (ng/\mu l) \times V_{tot} \ (\mu l) \times \frac{1 \ \mu g}{1000 \ ng}$$

Formula S1. Formula used for DNA encapsulation calculations. Initial DNA concentration before encapsulation in SUVs is known. After encapsulation and column filtration, the free DNA (eluate) is measured using NanoDrop, and extrapolated to the entire eluate volume. Subtracting this value from the initial DNA amount, the encapsulation efficiency is calculated.

$$\text{Noise signal (\%)} = 2^{-(Cq_{NTC} - Cq_{\text{lowest std concentration}})} \times 100$$

Formula S2. Formula used for background noise calculations. Using the difference in Cq (∆Cq) between the NTC and the lowest standard concentration, the noise percentage is calculated. For an acceptable qPCR result, the minimum ∆Cq is around 5, corresponding to a maximum noise percentage of 3%.
